# Q-rung orthopair fuzzy framework for multimodal MRI image fusion and U-Net-based segmentation for brain tumor detection

**DOI:** 10.3389/frai.2026.1840304

**Published:** 2026-10-09

**Authors:** Felix Augustin, Deva Nithyanandham, Mahesh Kumar C. V., David Raj M., Saravanakumar Ramasamy, Saraswathi D., Pandiyaraju V

**Affiliations:** 1 Department of Mathematics, School of Advanced Sciences, Vellore Institute of Technology, Chennai, India; 2 MSU-BIT-SMBU Joint Research Center of Applied Mathematics, Shenzhen MSU-BIT University, Shenzhen, China; 3 School of Mathematics and Statistics, Beijing Institute of Technology, Beijing, China; 4 Centre for Human Movement Analytics, School of Computer Science and Engineering, Vellore Institute of Technology, Chennai, India; 5 School of Computer Science and Engineering, Vellore Institute of Technology, Chennai, India

**Keywords:** brain tumor segmentation, medical image fusion, q-rung orthopair fuzzy set, t-norm and t-conorm, U-Net

## Abstract

**Introduction:**

Medical image analysis is a significant research domain in contemporary healthcare systems, which helps to accurately diagnose diseases and support clinical decision-making. This domain consists of important operations, including image enhancement, fusion, segmentation, morphological processing, and denoising, which assist in the effective understanding of complex biomedical information. Multimodal image fusion and segmentation are particularly important among these processes, as they enable the integration of complementary diagnostic information and the clear interpretation of clinically meaningful structures. However, medical images often consist of uncertainty, noise, and incomplete pixel information, creating major challenges for conventional analytical techniques.

**Methods:**

To address these challenges, the present study introduces a q-rung orthopair fuzzy set (qROFS)-based framework for multimodal magnetic resonance imaging (MRI) fusion, subsequently a U-Net-based segmentation architecture for brain tumor detection. To effectively handle uncertainty and preserve important image information, the fusion strategy is designed based on q-rung orthopair fuzzy Hamacher t-norm and t-conorm operators to integrate complementary information in four different MRI modalities, including T1, T1ce, T2, and Flair. The resultant fused images are subsequently processed through a U-Net architecture to obtain precise tumor segmentation.

**Results and discussion:**

Experiments conducted on the BraTS 2020 benchmark dataset demonstrate the effectiveness of the proposed framework. Across the four MRI modalities (T1, T1ce, T2, and Flair), the proposed fusion framework attains the best quantitative performance when compared to state-of-the-art methods with an SSIM of 0.9271, an MSSIM of 0.9481, a PSNR of 24.7130 dB, a low AMBE of 6.6779, a Correlation coefficient of 0.9583, an MI of 1.4097, and a UQI of 0.9767. A comparative evaluation with existing fuzzy set-based fusion models further assures the effectiveness of the proposed qROFS-based framework for multimodal MRI image fusion. Furthermore, the U-Net-based segmentation model achieves excellent performance, with an accuracy of 98.96%, a precision of 98.95%, a sensitivity of 98.94%, and a specificity of 99.65%.

## 1 Introduction

Image processing is a rapidly growing research field that greatly benefits various domains of science and technology like medicine, traffic signals, surveillance, etc. Its operations like fusion, enhancement, segmentation, noise removal, etc., help in handling numerous real-time applications. Among these fields, image fusion plays an important role in the medical field. Image fusion is the process of integrating the features or specifications from different images into an image to determine an informative and accurate image. Medical image fusion is one of the significant applications of image fusion. The medical image fusion with reduced distortion aims to integrate the different images into a single image with clear information and visual efficiency. However, medical images often fail to expose the pixel details due to uncertainty and vagueness. To address such situations, the theory of fuzzy logic has been applied to successfully enhance medical images.

The fuzzy set was initially explored by Zadeh (1965), and it helps to represent the uncertain element by assigning membership grades (μ). In fuzzy sets, the non-membership grade of the element, which is known as the fuzzy complement, is considered simply 1−μ. However, human intuition can assign the non-membership grade to the element independently. Based on this focus, Atanassov (1986) defined the notion of the intuitionistic fuzzy set (IFS) for assigning the non-membership grades (ν) to the elements independently, or not corresponding to the degree of membership grades. In IFS, the non-membership grades are not always 1−μ, and sometimes, the elements may have hesitancy grades π. It also satisfies the condition μ+ν+π = 1. These parameters aid in effectively addressing the uncertainty in real-world problems. Sometimes, the condition of IFS μ+ν>1 due to the hesitancy grade. To overcome these limitations, Pythagorean fuzzy sets (PFS) (Yager, 2013), Fermatean fuzzy sets (FFS) (Senapati and Yager, 2020), and q-rung orthopair fuzzy sets (qROFS) (Yager, 2016) have been defined. This idea of IFS has been widely applied in the discipline of image processing to remove the uncertainty in the images or to improve low-contrast images. Gupta et al. (1977) and Yager (1980) proposed new versions of the fuzzy complement functions to generate non-membership degrees in fuzzy sets, and it also helps to generate hesitancy grades.

Recently, many researchers have utilized the idea of the intuitionistic fuzzy generator (IFG) for the image enhancement process. Chaira (2020) developed an innovative IFG function-based model to enhance mammogram images by transforming them into interval type-2 fuzzy images. Later, Chaira (2021) applied an intuitionistic fuzzy clustering model using the IFG to improve mammogram image quality further. Jebadass and Balasubramaniam (2022b) employed Yager's IFG for a low-light image enhancement framework combined with the histogram equalization (HE) technique. Additionally, Jebadass and Balasubramaniam utilized Yager's and Chaira's (Chaira, 2020) IFG to develop the interval-valued IFS (IVIFS) based image enhancement model in Jebadass and Balasubramaniam (2022a) and Jebadass and Balasubramaniam (2024), respectively. Nandhini et al. (2024) introduced a new IFG model for clustering-based segmentation to enhance brain images. Most recently, Selvam et al. (2024) implemented a novel IFG combined with contrast-limited adaptive HE (CLAHE) for enhancing low-contrast images and demonstrated the superiority of the results over previous techniques. Chinnappan and Sundaram (2025) developed a video enhancement model using a new IFG and HE technique. Selvam and Sundaram (2025) proposed a new IVIFS-based image enhancement model to enhance the low-light images using their ground truth. Maheshkumar et al. (2025) derived a generalized Sugeno's IFG to propose a low-light enhancement method with a new searching algorithm called BestIFI for optimizing the parameters. Ragavendirane and Dhanasekar (2025) developed a new IFG based mage enhancement model to improve the visual quality of low-light images using the HE-Retinex method. Selvam et al. (2026) proposed a novel sand-dust image enhancement model by combining an IFG, gray world algorithm, and dehazing method. Further, Selvam and Sundaram (2026) designed an underwater image enhancement approach by applying IFG with the gray world algorithm and CLAHE approaches. As a novel approach, Mahesh Kumar et al. (2026) proposed a bipolar fuzzy set-based adaptive model to effectively enhance low-light images.

Various researchers have also utilized the IFG to design image fusion models. Balasubramaniam and Ananthi (2014) proposed a novel method to fuse images by applying minimum and maximum operators. To generate the intuitionistic fuzzy image (IFI), Chiara's IFG (Chaira, 2012) has been utilized. Jebadass and Balasubramaniam (2023) extended Chaira's IFG to design an image fusion method using an interval-valued type-2 fuzzy set approach. Tirupal et al. (2017) constructed an image fusion model using Sugeno's IFG for multimodal medical image fusion (MMIF) process. For the experiment, the authors considered the T1-weighted MR and MRA images. Palanisami et al. (2022) developed an MMIF model using Sugeno's IFG, and the Gaussian filter was applied for two-scale decomposition to determine the base layer and detail layer. For the experimental purpose of this MMIF, the authors considered four types of databases such as CT and MRI, MR-Gad and MR-T2, MR-PD and MR-T2, and MR-T1 and MR-T2. Haribabu and Guruviah (2023b) designed an enhanced MMIF using a new IFG and intuitionistic fuzzy cross-correlation measure. Also, Haribabu and Guruviah (2023a) proposed a new MMIF model to combine different modalities of medical images. For the experiment, the authors utilized CT and MRI, T1-weighted MR and MRA, T1-MR and T2-MR, MR-T2 and SPECT, and MRI and PET images. Recently, Haribabu and Guruviah (2025) framed a MMIF model using FFS and the whale optimization technique for optimizing the parameters in the generating function during the fusion process.

U-Net is one of the efficient network model which greatly helps in the image segmentation process. Its architecture serves as an efficient tool to segment images to deal with many real-world problems in numerous domains, including medicine, agriculture, electronics, etc. Recently, researchers have widely applied the U-Net model for the process of image segmentation. Huang et al. (2021) designed a model called Group Cross-Channel Attention Residual U-Net (GCAU-Net) to enhance segmentation of brain tumors by effectively utilizing fine low-level tumor details. In this, the preprocessing steps include iterative background removal and image normalization. GCAU-Net incorporates a detail-recovering path for extracting low-level multiscale features and a Group Cross-Channel Attention (GCA) Module to highlight important feature groups. Additionally, a multiscale input Path captures contextual information. On the BraTS 2017 and BraTS 2018 datasets, GCAU-Net determined dice scores of 87.2%/79.6%/78.1% (WT/TC/ET) and 90.9%/84.5%/81.3%, respectively, representing significant improvements over baseline methods. To enhance scalability in brain tumor segmentation (BTS), Latif et al. (2021) introduced a Multi-Inception-U-Net model as an extension of the U-Net architecture for pixel-wise classification of tumor slices. In these experiments, extensive experiments were conducted on the BraTS 2015, 2017, and 2019 datasets, demonstrating excellent performance in tumor core (TC), whole tumor (WT), and enhancing tumor (ET) region segmentation, validating the model as a robust solution for segmentation tasks. Aghalari et al. (2021) developed an enhanced U-Net model for BTS by employing Two-Pathway-Residual blocks to extract both local and global features. With the BraTS 2018 dataset, the TPRED-UNet demonstrated segmentation scores of 89.76% (Dice), 89.19% (Sensitivity), 90.65% (PPV), and 99.87% (Specificity), while maintaining low computational costs compared to other methods. Luu and Park (2021) designed the nn-U-Net model with modifications such as axial attention, a larger network, and group normalization in the decoder. The model achieved dice scores of 93.19% (WT), 88.78% (TC), and 88.35% (ET) on unseen test data. Hu et al. (2021) proposed a hybrid segmentation model by combining U-Net and long short-term memory (LSTM) networks for efficient multimodal BTS. To address class imbalance, they also derived a composite loss function, which improved segmentation performance. With the BraTS 2017 dataset, the model achieved Dice scores of 0.91, 0.82, and 0.80 for WT, TC, and ET, respectively. Saha et al. (2021) developed a Multi-Pathway U-Net model based on residual networks and skip connections to precisely delineate glioma sub-regions from multimodal MRI scans. Jena et al. (2023) conducted a systematic review on the influence of network depth within the U-Net model, focusing on the balance between segmentation accuracy and computational cost. Their findings indicate that the reduction of architectural depth resulted in low computational cost while maintaining effective segmentation performance. With the BraTS 2017 and 2019 datasets, the model yields Dice scores ranging from 0.8866 to 0.8911. Allah et al. (2023) proposed the Edge U-Net model to enhance tumor localization through the fusion of boundary-aware MRI data with global contextual cues. To further enhance segmentation accuracy, a boundary-sensitive loss function was derived. Testing on a dataset consisting of 3064 T1ce images obtained Dice coefficients of 87.28% for pituitary tumors, 88.80% for meningioma, and 91.76% for glioma, showing its clinical applicability. Fang and Wang (2023) designed a multi-input U-Net model to effectively enhance BTS. By integrating mask-guided inputs, the model enables precise spatial localization of tumor regions, while ensuring the extraction of discriminative features across varying receptive fields based on multi-scale convolutional kernels. To reduce computational burden, dimension compression methods are considered without compromising important structural information. Ablation experiments on the BraTS 2018 dataset ensure the strength of these components, achieving precision values of 0.92 for WT and 0.90 for TC. Raza et al. (2023) developed a robust three-dimensional BTS model using a hybrid deep learning architecture, dResU-Net, which combines a deep residual network as the encoder with a U-Net-style decoder. This design effectively helps address vanishing gradient issues while enabling the subsequent exploitation of fine-grained and abstract feature representations. Residual shortcut pathways maintain low-level structural information, whereas skip connections improve convergence speed during the training process. Experiments on the BraTS 2020 dataset demonstrated Dice scores of 0.8357 for TC, 0.8004 for ET, and 0.8660 for WT. Further, an evaluation on an independent cohort of 50 patients from the BraTS 2021 dataset obtained Dice values of 0.8400 (TC), 0.8221 (ET), and 0.8601 (WT), ensuring the model's superior segmentation performance compared to existing models.

Most recently, Liu et al. (2025) designed a new U-shaped segmentation method called CSWin-U-Net that combines the U-Net model with a CSWin self-attention mechanism to ease vertical and horizontal stripes self-attention. To evaluate the model, various datasets including skin lesions, cardiac MRI, and multi-organ CT images were segmented, and these evaluations demonstrate that the model maintains low complexity and high accuracy (91.40%) in the segmentation process. Munia et al. (2025) proposed a new model called an attention-guided hierarchical fusion U-Net model to segment uncertainty-driven medical images. Also, an uncertainty-aware version was proposed by integrating a decoder and encoder attention fusion module on the decoder and encoder sides of the U-Net architecture, respectively. Additionally, this model was evaluated with eleven existing models on well-known datasets and obtained 82.51% of Dice, 70.33% of IoU, 96.89% of accuracy, 80.34% of sensitivity, and 98.60% of specificity.

From the detailed review of existing works, it is observed that the IFG greatly helps to enhance and fuse the medical images. Also, the U-Net significantly aids in segmenting the medical images with better accuracy. Based on a review of existing works, the motivation of the study is discussed below.

### 1.1 Motivation of the work

MMIF and segmentation constitute essential components of contemporary clinical imaging systems, facilitating precise diagnosis and informed therapeutic decision-making. The integration of complementary information from heterogeneous imaging sources, such as computed tomography (CT), positron emission tomography (PET), magnetic resonance imaging (MRI), and single-photon emission computed tomography (SPECT), significantly enhances the visualization of anatomical and pathological characteristics. This combined process is significant for applications, including tumor localization, organ delineation, and longitudinal disease evaluation. However, medical imaging information often contains noise, sensor inaccuracies, and environmental disturbances, which introduce incomplete pixel information and uncertainty, complicating image representation.

Conventional models often present limited robustness when processed with such uncertainties, creating the need for more adaptive and intelligent frameworks. In such conditions, fuzzy logic-based models, including IFS, PFS, FFS, and qROFS, offer a mathematically rigorous foundation for comprising ambiguity and imprecision in imaging data. Integration of these sets with appropriate aggregation approaches, such as t-norm and t-conorm operators, yields effective fusion performance and improved visual accuracy.

For segmentation processes, the U-Net architecture has demonstrated noteworthy capability in accurately isolating pathological regions from complex medical images. However, conventional models frequently encounter challenges when handling ambiguous boundary regions and noisy pixel distributions. Integrating fuzzy logic representations within the U-Net model offers an effective approach to model uncertainty during pixel-level classification, leading to more interpretable, stable, and clinically reliable segmentation outputs.

The ongoing development of fusion and segmentation approaches is significantly necessary for the enhanced structural integrity, clarity, and diagnostic reliability of images. Such improvements play a significant role in assisting clinicians to address imaging limitations, ultimately contributing to enhanced patient treatment planning, diagnosis, and clinical outcomes.

### 1.2 Aim and objectives

The main aim of the present work is to design and implement a robust MMIF framework based on qROFS, followed by a U-Net-based segmentation model for effective BTS.

**Objectives:**

(i) To design a qROF framework for multimodal MRI image fusion to enhance clarity and tumor detection in the BraTS 2020 dataset.
(ii) To generate a qROF image to enhance the visual quality of the medical images.
(iii) To develop a fusion rule using qROF Hamacher t-norm and t-conorm operators to retain critical tumor-related details.
(iv) To employ the U-Net for segmentation and improved brain tumor analysis from the fused images.

### 1.3 Contribution

(i) **Novel qROF image:** A qROF image framework is introduced using an IFG based on Yager's functions for modeling uncertainty effectively.
(ii) **Fusion of multiple images:** A fusion model is designed to fuse four MRI images for advancing beyond traditional two-image fusion techniques.
(iii) **qROF Hamacher t-norm aggregation:** qROF Hamacher t-norm and t-conorm operators are applied to ensure robust image fusion with minimal loss of essential details.
(iv) **U-Net based segmentation:** The U-Net architecture is applied to segment fused images for determining precise tumor localization and analysis.
(v) **Comparative analysis:** The proposed method is compared with several existing image fusion frameworks including the method of simple average method (SAM), multi-resolution singular value decomposition (MSVD) (Naidu, 2011), principal component analysis (PCA), image fusion using intuitionistic fuzzy set (IFIFS) (Balasubramaniam and Ananthi, 2014), cross bilateral filter (CBF) (Shreyamsha Kumar, 2015), fast filtering image fusion (FFIF) (Zhan et al., 2017), guided image filter and image statistics (GIFS) (Bavirisetti et al., 2017), convolutional sparsity-based morphological component analysis (CSMCA) (Liu et al., 2019), and Gaussian of difference (GD) (Kurban, 2023).
(vi) **Comparison with various fuzzy sets:** A comparative study is performed using IFS, PFS, and FFS with qROFS in the proposed image fusion model to show the efficiency of the qROFS-based framework.

The overall structure of the manuscript is as follows: In Section 1, the introduction and literature review of the study are given. In Section 2, the fundamental notions are discussed to support the present study. In Section 3, the proposed framework is explored. In Section 4, the experimental analysis of the proposed model is discussed with comparative and quantitative studies. Finally, the conclusion of the present study is given in Section 5.

## 2 Preliminaries

A fuzzy set Ã (Zadeh, 1965) of a universal set *X* is defined as Ã = {(*x*, μ_Ã_(*x*))|*x*∈*X*}, where μ_Ã_(*x*):*X* → [0, 1] represents the membership function of Ã. An IFS $\tilde{B}$ (Atanassov, 1986) is defined as $\tilde{B}=\left\{\left(x,\mu_{\tilde{B}}\left(x\right),\gamma_{\tilde{B}}\left(x\right)\right)|\;x\in X\right\}$, where $\mu_{\tilde{B}}\left(x\right):X\to \left[0,1\right]$ and $\gamma_{\tilde{B}}\left(x\right):X\to \left[0,1\right]$ represent the membership and non-membership functions of $\tilde{B}$ such that $0\leq \mu_{\tilde{B}}\left(x\right)+\gamma_{\tilde{B}}\left(x\right)\leq 1$.

A fuzzy complement is defined as a mapping *c*:[0, 1] → [0, 1]. Then, the set $\tilde{B}=\left\{\left(x,\mu_{\tilde{B}}\left(x\right),c\left(\mu_{\tilde{B}}\left(x\right)\right)\right)|\;x\in X\right\}$ is not always an IFS.

Sometimes, the sum of membership and non-membership functions $\mu_{\tilde{B}}\left(x\right)+\gamma_{\tilde{B}}\left(x\right)$ may exceed 1 as both $\mu_{\tilde{B}}\left(x\right)$ and $\gamma_{\tilde{B}}\left(x\right)$ are mapping to [0, 1]. To overcome this issue, the notion of the PFS was developed by Yager (2013) as an extension of the IFS. It is denoted by $\tilde{P}=\left\{\left(x,\mu_{\tilde{P}}\left(x\right),\gamma_{\tilde{P}}\left(x\right)\right)|\;x\in X\right\}$, where $\mu_{\tilde{P}}\left(x\right),\gamma_{\tilde{P}}\left(x\right):X\to \left[0,1\right]$ such that $0\leq \mu_{\tilde{P}}^{2}\left(x\right)+\gamma_{\tilde{P}}^{2}\left(x\right)\leq 1$. In PFS, the hesitancy grade can be defined as $\pi_{\tilde{P}}\left(x\right)=\sqrt{1-\mu_{\tilde{B}}^{2}\left(x\right)-\gamma_{\tilde{B}}^{2}\left(x\right)}$ and it is mapping to [0, 1]. Similarly, FFS has the restriction $0\leq \mu_{\tilde{P}}^{3}\left(x\right)+\gamma_{\tilde{P}}^{3}\left(x\right)\leq 1$.

The PFS and FFS often fail to hold the above-mentioned restriction. To handle the problem, the generalized version of IFS called qROFS was introduced by Yager (2016). It is defined as $\tilde{Q}=\left\{\left(x,\mu_{\tilde{Q}}\left(x\right),\gamma_{\tilde{Q}}\left(x\right)\right)|\;x\in X\right\}$, where $\mu_{\tilde{Q}}\left(x\right),\gamma_{\tilde{Q}}\left(x\right):X\to \left[0,1\right]$ such that $0\leq \mu_{\tilde{Q}}^{q}\left(x\right)+\gamma_{\tilde{Q}}^{q}\left(x\right)\leq 1$. Here, *q* is a positive integer. In qROFS, the hesitancy grade can be defined as $\pi_{\tilde{Q}}\left(x\right)=\sqrt[{q}]{1-\mu_{\tilde{Q}}^{q}\left(x\right)-\gamma_{\tilde{Q}}^{q}\left(x\right)}$ and it is mapping to [0, 1].

A function *T*:[0, 1] × [0, 1] → [0, 1] is defined as a t-norm if it holds the following conditions:

(i) *T*(*x*, 0) = 0 and *T*(*x*, 1) = *x*, for all *x*∈[0, 1]
(ii) *T*(*x, y*) = *T*(*y, x*), for all *x*∈[0, 1]
(iii) *T*(*x, T*(*y, z*)) = *T*(*T*(*x, y*), *z*), for all *x, y, z*∈[0, 1]
(iv) *x* ≤ *z, y* ≤ *w* ⇒ *T*(*x, y*) ≤ *T*(*z, w*), for all *x, y, z*∈[0, 1]

A function *U*:[0, 1] × [0, 1] → [0, 1] is defined as a t-conorm if it holds the following axioms:

(i) *U*(*x*, 0) = *x* and *U*(*x*, 1) = 1, for all *x*∈[0, 1]
(ii) *U*(*x, y*) = *U*(*y, x*), for all *x*∈[0, 1]
(iii) *U*(*x, U*(*y, z*)) = *U*(*U*(*x, y*), *z*), for all *x, y, z*∈[0, 1]
(iv) *x* ≤ *z, y* ≤ *w* ⇒ *U*(*x, y*) ≤ *U*(*z, w*), for all *x, y, z*∈[0, 1]

Hamacher (1975) defined the Hamacher product and sum as extensions of t-norms and t-conorms, respectively. The Hamacher t-norm ⊗ and the Hamacher t-conorm ⊕ are expressed as follows:

-
$T\left(x,y\right)=x⊗y=\frac{xy}{\delta +\left(1-\delta \right)\left(x+y-xy\right)},\;\beta >0$

-
$U\left(x,y\right)=x⊕y=\frac{x+y-xy-\left(1-\delta \right)xy}{1-\left(1-\delta \right)\left(xy\right)},\;\delta >0$

Let ${\tilde{Q}}_{1}=\left\{\left(x,\mu_{{\tilde{Q}}_{1}}\left(x\right),\gamma_{{\tilde{Q}}_{1}}\left(x\right)\right)|\;x\in X\right\}$ and ${\tilde{Q}}_{2}=\left\{\left(y,\mu_{{\tilde{Q}}_{2}}\left(y\right),\gamma_{{\tilde{Q}}_{2}}\left(y\right)\right)|\;y\in X\right\}$ be any two qROFS. Then, Hamacher t-norm and t-conorm functions (Komal, 2023) are defined as:


$$\begin{array}{l} T_{\delta }^{H}\left(x,y\right)={\left(\frac{x^{q}y^{q}}{\delta +\left(1-\delta \right)\left(x^{q}+y^{q}-x^{q}y^{q}\right)}\right)}^{1/q},\;\delta >0 \end{array}$$ (1)

$$\begin{array}{l} U_{\delta }^{H}\left(x,y\right)={\left(\frac{x^{q}+y^{q}-x^{q}y^{q}-\left(1-\delta \right)x^{q}y^{q}}{1-\left(1-\delta \right)x^{q}y^{q}}\right)}^{1/q},\;\delta >0 \end{array}$$ (2)

### 2.1 Fuzzy sets of an image

Images naturally exhibit various characteristics that can introduce fuzziness. The question arises: what makes an image fuzzy? Several image properties, such as edges and intensity levels, are inherently imprecise. This fuzziness may result from limitations in imaging devices, poor lighting conditions, or the innate ambiguity in the captured image. Since the intensity values within a pixel can embody uncertainty, this is treated as fuzziness throughout this work. This uncertainty necessitates the transformation of images into the fuzzy domain, which allows the membership function to span the entire intensity range. The process of fuzzification involves quantifying and normalizing pixel intensity values.

For an image of dimensions *M*×*N* with *L* levels of grayness, it can be viewed as an *M*×*N* array of fuzzy singletons representing pixel intensities. More details on this concept can be found in Chaira and Ray (2017). A fuzzy image (*I*_*F*_) derived from an image (*I*) is mathematically represented as *I*_*F*_:*M*×*N*→*G* → [0, 1].

Here, *I*(*i, j*) = *g* denotes the intensity value of the (*i, j*)^th^ pixel, with *G* = {0, 1, 2, …, *L*−1} representing the set of positive integers corresponding to the gray levels of the image. The membership function μ_*F*_(*g*) defines the membership of *g* in the fuzzy set, as given in Equation 3:


$$\begin{array}{l} \mu_{F}\left(I\left(i,j\right)\right)=\frac{g-g_{\text{min}}}{g_{\text{max}}-g_{\text{min}}} \end{array}$$ (3)

where *g*_min_ and *g*_max_ are the lowest and highest intensity values of gray levels in the image, respectively. Consequently, the fuzzy representation of the image *I* is given by: *I*_*F*_ = {*I*(*i, j*), μ_*F*_(*I*(*i, j*)) | 0 ≤ *I*(*i, j*) ≤ *L*−1, 0 ≤ μ_*F*_(*I*(*i, j*)) ≤ 1, 1 ≤ *i* ≤ *M*, 1 ≤ *j* ≤ *N*}.

### 2.2 Construction of intuitionistic, Pythagorean, and Fermatean fuzzy sets

IFG is utilized to construct the IFS. A function ϕ:[0, 1] → [0, 1] is said to be an IFG if it satisfy Equation 4


$$\begin{array}{l} \phi \left(x\right)\leq 1-x,\;\forall x\in \left[0,1\right] \end{array}$$ (4)

and ϕ(0) ≤ 1 and ϕ(1) ≤ 0.

Fuzzy complement is utilized in the construction of IFG. The function ν is said to be fuzzy complement if and only if there exists a continuous function *n* such that *n*(0) = 0 and ν can be represented as ν(*x*) = *n*^−1^(*n*(1)−*n*(*x*)). To construct the Yager's IFG (Equation 5), let *n*(*x*) = *x*^β^, where *n*(0) = 0 and *n*(1) = 1. From this


$$\begin{array}{l} \gamma \left(x\right)={\left(1-x^{\beta }\right)}^{1/\beta },\beta \geq 0 \end{array}$$ (5)

The condition of IFG does not satisfy when β>1, so the IFG is γ(*x*) = (1−*x*^β^)1/β, 0 ≤ β ≤ 1. Hence, the IFS can be defined as $\tilde{B}=\left\{\left(x,\mu_{\tilde{B}}\left(x\right),{\left(1-{\left(\mu_{\tilde{B}}\left(x\right)\right)}^{\beta }\right)}^{1/\beta }\right)|\;x\in X\right\}$ and the hesitancy degree defined as $\pi_{\tilde{B}}\left(x\right)=1-\mu_{\tilde{B}}\left(x\right)-{\left(1-{\left(\mu_{\tilde{B}}\left(x\right)\right)}^{\beta }\right)}^{1/\beta }$.

In general, the IFS is defined as $\tilde{B}=\{(x,\mu_{\tilde{B}}(x),\gamma_{\tilde{B}}(x)\;x\in X\}$ and the hesitancy degree defined as $\pi_{\tilde{B}}\left(x\right)=1-\mu_{\tilde{B}}\left(x\right)-\gamma_{\tilde{B}}\left(x\right)$.

The construction of PFS (Yager, 2013) and FFS (Senapati and Yager, 2020) in image processing is the same as the construction of IFS in image processing. The difference among IFS, PFS, and FFS in image processing lies in the degree of hesitancy they incorporate. Hesitancy degrees of PFS and FFS are calculated as $\pi_{\tilde{P}}\left(x\right)=\sqrt{1-{\left(\mu_{\tilde{B}}\left(x\right)\right)}^{2}-{\left(\gamma_{\tilde{B}}\left(x\right)\right)}^{2}}$ and $\pi_{\tilde{F}}\left(x\right)=\sqrt[{3}]{1-{\left(\mu_{\tilde{B}}\left(x\right)\right)}^{3}-{\left(\gamma_{\tilde{B}}\left(x\right)\right)}^{3}}$.

### 2.3 Construction of Intuitionistic, Pythagorean, and Fermatean Fuzzy Images

Let us consider the IFS, PFS, or FFS $\tilde{B}=\left\{\left(x,\mu_{\tilde{B}}\left(x\right),\gamma_{\tilde{B}}\left(x\right)\right)|\;x\in X\right\}$. The given image *I*(*i, j*) is converted to fuzzy image *I*_*F*_(*i, j*) with the help of membership function $\mu_{\tilde{B}}$, where *I*(*i, j*) is the (*i, j*)*th* pixel of the image *I*, *I*_*F*_(*i, j*) is the (*i, j*)*th* pixel in the fuzzy image. The non-membership image is denoted as $\gamma_{\tilde{B}}\left(I_{F}\left(i,j\right)\right)$, where $\gamma_{\tilde{B}}\left(I_{F}\left(i,j\right)\right)$ is the (*i, j*)*th* pixel in the non membership image.

The hesitation image $\pi_{\tilde{B}}\left(I_{F}\left(i,j\right)\right)$ for IFS (Atanassov, 1986) is calculated as $\pi_{\tilde{B}}\left(I_{F}\left(i,j\right)\right)=1-\mu_{\tilde{B}}\left(I_{F}\left(i,j\right)\right)-\gamma_{\tilde{B}}\left(I_{F}\left(i,j\right)\right)$. Similarly, the hesitation image $\pi_{\tilde{P}}\left(I_{F}\left(i,j\right)\right)$ for PFS and FFS can be calculated as $\pi_{\tilde{P}}\left(I_{F}\left(i,j\right)\right)=\sqrt{1-{\left(\mu_{\tilde{B}}\left(I_{F}\left(i,j\right)\right)\right)}^{2}-{\left(\gamma_{\tilde{B}}\left(I_{F}\left(i,j\right)\right)\right)}^{2}}$ and $\pi_{\tilde{F}}\left(I_{F}\left(i,j\right)\right)=\sqrt[{3}]{1-{\left(\mu_{\tilde{B}}\left(I_{F}\left(i,j\right)\right)\right)}^{3}-{\left(\gamma_{\tilde{B}}\left(I_{F}\left(i,j\right)\right)\right)}^{3}}$, respectively.

Based on these computations, the intuitionistic fuzzy image (IFI) *I*_*B*_(*i, j*), Pythagorean fuzzy image (PFI) *I*_*P*_(*i, j*), and Fermatean fuzzy image (FFI) *I*_*FF*_(*i, j*) can be constructed as $I_{B}\left(i,j\right)=\mu_{\tilde{B}}\left(I_{F}\left(i,j\right)\right)+\pi_{\tilde{B}}\left(I_{F}\left(i,j\right)\right)$, $I_{P}\left(i,j\right)=\mu_{\tilde{B}}\left(I_{F}\left(i,j\right)\right)+\pi_{\tilde{P}}\left(I_{F}\left(i,j\right)\right)$ and $I_{FF}\left(i,j\right)=\mu_{\tilde{B}}\left(I_{F}\left(i,j\right)\right)+\pi_{\tilde{F}}\left(I_{F}\left(i,j\right)\right)$, respectively.

## 3 Proposed framework

In this part, the construction of qROFI, the fusion rule, and the algorithm of the proposed qROF framework and U-Net approach for MMIF and segmentation are discussed.

### 3.1 Construction of q-rung orthopair fuzzy image

The construction of qROFS in image processing is the same as that of the construction of IFS, PFS, and FFS. The qROFS is defined as $\tilde{Q}=\left\{\left(x,\mu_{\tilde{Q}}\left(x\right),\gamma_{\tilde{Q}}\left(x\right)\right)|\;x\in X\right\}$. Here, the hesitancy degree is calculated as $\pi_{\tilde{Q}}\left(x\right)=\sqrt[{q}]{1-{\left(\mu_{\tilde{Q}}\left(x\right)\right)}^{q}-{\left(\gamma_{\tilde{Q}}\left(x\right)\right)}^{q}}$.

The membership image $\mu_{\tilde{Q}}\left(I_{F}\left(i,j\right)\right)$ and non-membership image $\gamma_{\tilde{Q}}\left(I_{F}\left(i,j\right)\right)$ is calculated as in Subsection 2.3. The Hesitation image is calculated as shown in Equation 6.


$$\begin{array}{l} \pi_{\tilde{Q}}\left(I_{F}\left(i,j\right)\right)=\sqrt[{q}]{1-{\left(\mu_{\tilde{Q}}\left(I_{F}\left(i,j\right)\right)\right)}^{q}-{\left(\gamma_{\tilde{Q}}\left(I_{F}\left(i,j\right)\right)\right)}^{q}} \end{array}$$ (6)

Then, the qROF image (qROFI) *I*_*Q*_(*i, j*) is constructed as given in Equation 7.


$$\begin{array}{l} I_{Q}\left(i,j\right)=\mu_{\tilde{Q}}\left(I_{F}\left(i,j\right)\right)+\pi_{\tilde{Q}}\left(I_{F}\left(i,j\right)\right) \end{array}$$ (7)

where *I*_*Q*_(*i, j*) is the (*i, j*) pixel in the qROFI.

In this construction, an arbitrary *q* may not provide a better fusion result for all the images. It may differ for each image based on the information of the pixels. The selection of an optimal *q* leads to a new research problem. However, in this work, an arbitrary *q* is fixed after several experiments.

### 3.2 Fusion rule

The fusion rule is constructed to fuse all four images using t-norm and t-conorm functions, which is represented as follows:


$$I_{f}=\{\begin{array}{ll} T(T(I^{1ab},I^{2ab}),T(I^{3ab},I^{4ab})) & \text{count(black)}>\text{count(white)}\\ U(U(I^{2ab},I^{2ab}),U(I^{3ab},I^{4ab})) & \text{count(black)}<\text{count(white)}\\ \frac{I^{1ab}+I^{2ab}+I^{3ab}+I^{4ab}}{4} & \text{Otherwise} \end{array}$$ (8)

where *I*^*kab*^ represents the separated block of *a*th row and *b*th column of *k*th modal.

### 3.3 Algorithm of proposed model

The proposed model is constructed as a sequential process of two stages, in which stage-1 is designed with the qROF framework for MMIF and stage-2 is designed with the U-Net model for the segmentation process of fused images.

#### 3.3.1 Stage 1—Fusion process

**Step 1:** Input the four modality of MRI images *I*^*k*^(*i, j*), *k* = 1, 2, 3, 4.

The different modalities of MRI images can be considered as input images.

**Step 2:** Fuzzify the input images *I*^*k*^(*i, j*) to obtain the fuzzy images $I_{F}^{k}\left(i,j\right)$.

The input images can be fuzzified using Equation 9:


$$\begin{array}{l} I_{F}^{k}\left(i,j\right)=\frac{I^{k}\left(i,j\right)-I_{\min}}{I_{\max}-I_{\min}} \end{array}$$ (9)

where *I*_max_ and *I*_min_ represent the maximum and minimum intensities in the input image.

**Step 3:** Set β value to determine membership $\left(\mu_{\tilde{Q}}\left(I_{F}^{k}\left(i,j\right)\right)\right)$, non-membership $\left(\gamma_{\tilde{Q}}\left(I_{F}^{k}\left(i,j\right)\right)\right)$ and hesitancy $\left(\pi_{\tilde{Q}}\left(I_{F}^{k}\left(i,j\right)\right)\right)$ grades of the input images using Yager's IFG.

By fixing the parameter β value, the membership $\left(\mu_{\tilde{Q}}\left(I_{F}^{k}\left(i,j\right)\right)\right)$, non-membership $\left(\gamma_{\tilde{Q}}\left(I_{F}^{k}\left(i,j\right)\right)\right)$ and hesitancy $\left(\pi_{\tilde{Q}}\left(I_{F}^{k}\left(i,j\right)\right)\right)$ grades of qROFI $I_{Q}^{k}\left(i,j\right),\;k=1,2,3,4$ can be generated.

**Step 4:** Construct the qROFI using Step 4.

The qROFI can be constructed using the following Equation 10.


$$\begin{array}{l} I_{Q}^{k}\left(i,j\right)=\mu_{\tilde{Q}}\left(I_{F}^{k}\left(i,j\right)\right)+\pi_{\tilde{Q}}\left(I_{F}^{k}\left(i,j\right)\right) \end{array}$$ (10)

**Step 5:** Decompose each of qROFIs into *a*×*b* blocks

The qROF images can be decomposed into *a*×*b* blocks, where the block of *l*^*th*^ row and *m*^*th*^ column of the images are represented as *I*^1*lm*^, *I*^2*lm*^, *I*^3*lm*^ and *I*^4*lm*^.

**Step 6:** Fuse the different modalities of the qROFI.

The different modalities of the qROFI can be fused using the following fusion rule given in Equation 8. The fused qROFI can be represented as *I*_*Qf*_.

**Step 7:** Defuzzify the fused image to obtain the output image

The fused images can be defuzzified using Equation 11. It is considered the output image $I_{Q}^{*}$.


$$\begin{array}{l} I_{Q}^{*}=\left(\left(I_{\max}-I_{\min}\right)*I_{Qf}+I_{\min}\right) \end{array}$$ (11)

A visual flowchart of the fusion process is demonstrated in Figure 1.

**Figure 1:**
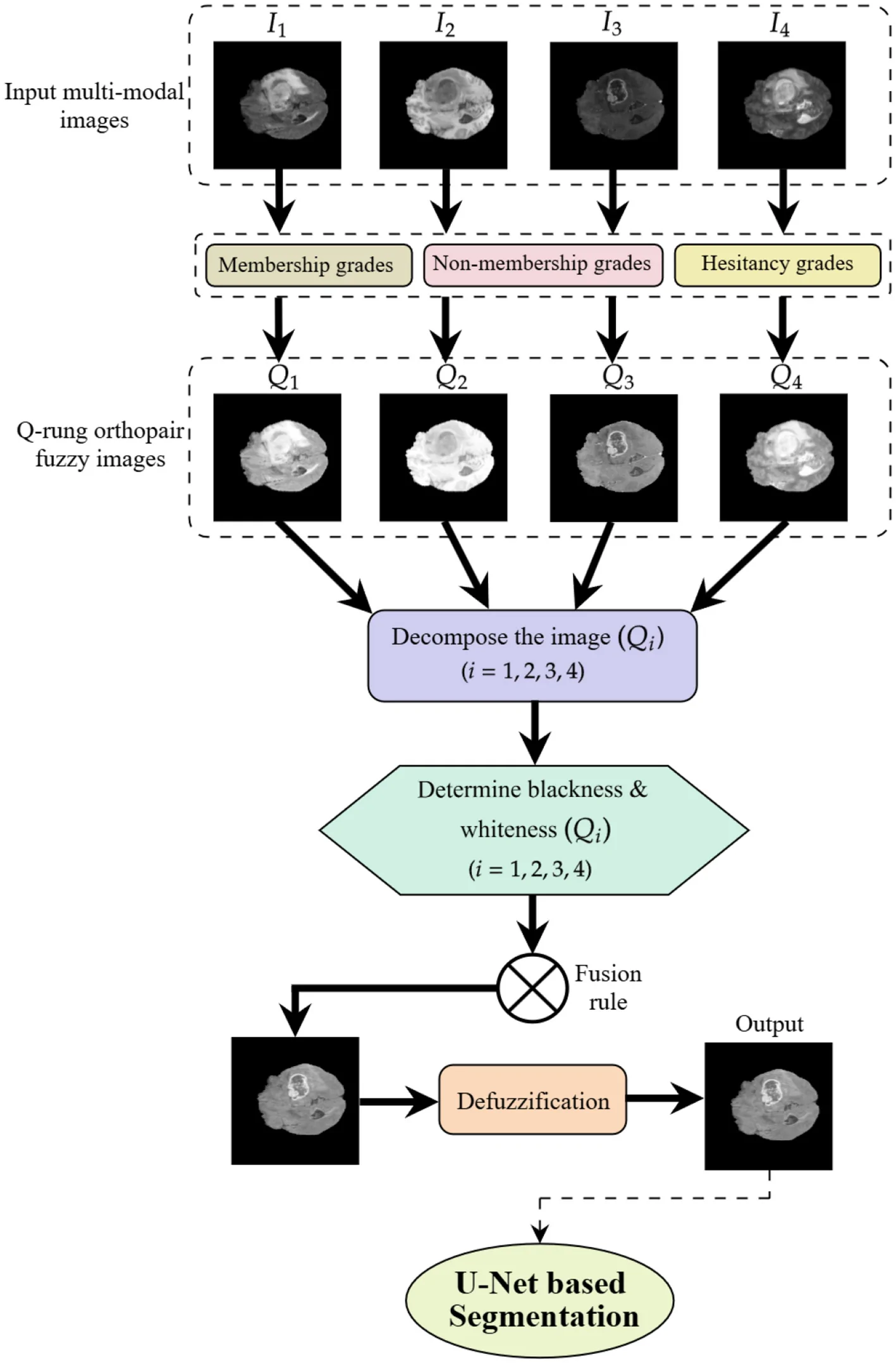
Work flow of fusion process (Stage 1).

#### 3.3.2 Stage 2—Segmentation process

**Input:** Fused image $I_{Q}^{*}$

**Output:** Segmented output image *I*_*S*_

**Step 1:** Preprocessing

1. Resize the fused image (input) $I_{Q}^{*}$ to a size of 128 × 128.
2. Normalize the pixel values to the range [0, 1] or standardize based on the mean and standard deviation of the dataset.

**Step 2:** Encoding path (contracting path)The encoder captures the image's contextual information using downsampling and feature extraction.

1. Convolution block: Apply two 3 × 3 convolution layers with ReLU activation and follow each convolution with batch normalization to stabilize training.
2. Downsampling: Use a 2 × 2 max pooling layer to reduce the spatial dimensions by half and double the number of feature channels after each pooling step.
3. Repeat the convolution and downsampling steps multiple times (four levels).

**Step 3:** BottleneckAt the bottleneck layer, perform two 3 × 3 convolutions with ReLU activation and apply batch normalization.

**Step 4:** Decoding path (expanding path)The decoder restores spatial resolution while combining low-level and high-level features.

1. Upsampling: Apply 2 × 2 transposed convolution (deconvolution) to upsample the feature map by a factor of 2
2. Skip Connections: Concatenate the upsampled feature map with the corresponding feature map from the encoder path (same spatial size) to retain fine details
3. Convolution Block: Apply two 3 × 3 convolution layers with ReLU activation, followed by batch normalization (optional)
4. Repeat the upsampling and convolution steps until the original spatial dimensions are restored.

**Step 5:** Final output layer

1. Apply a 1 × 1 convolution to reduce the number of feature channels to the number of classes.
2. Use a softmax (multi-class) or sigmoid (binary) activation function to output class probabilities for each pixel.

**Step 6:** Post-processing

1. Convert probability maps into binary masks or multi-class segmentation by thresholding.
2. Refine the output using morphological operations, if needed.

**Step 7:** Get the segmented image *I*_*S*_.

The pseudocode of the qROF image fusion framework for a slice is provided in Algorithm 1.

Algorithm 1 Pseudocode of qROF image fusion for a slice. 
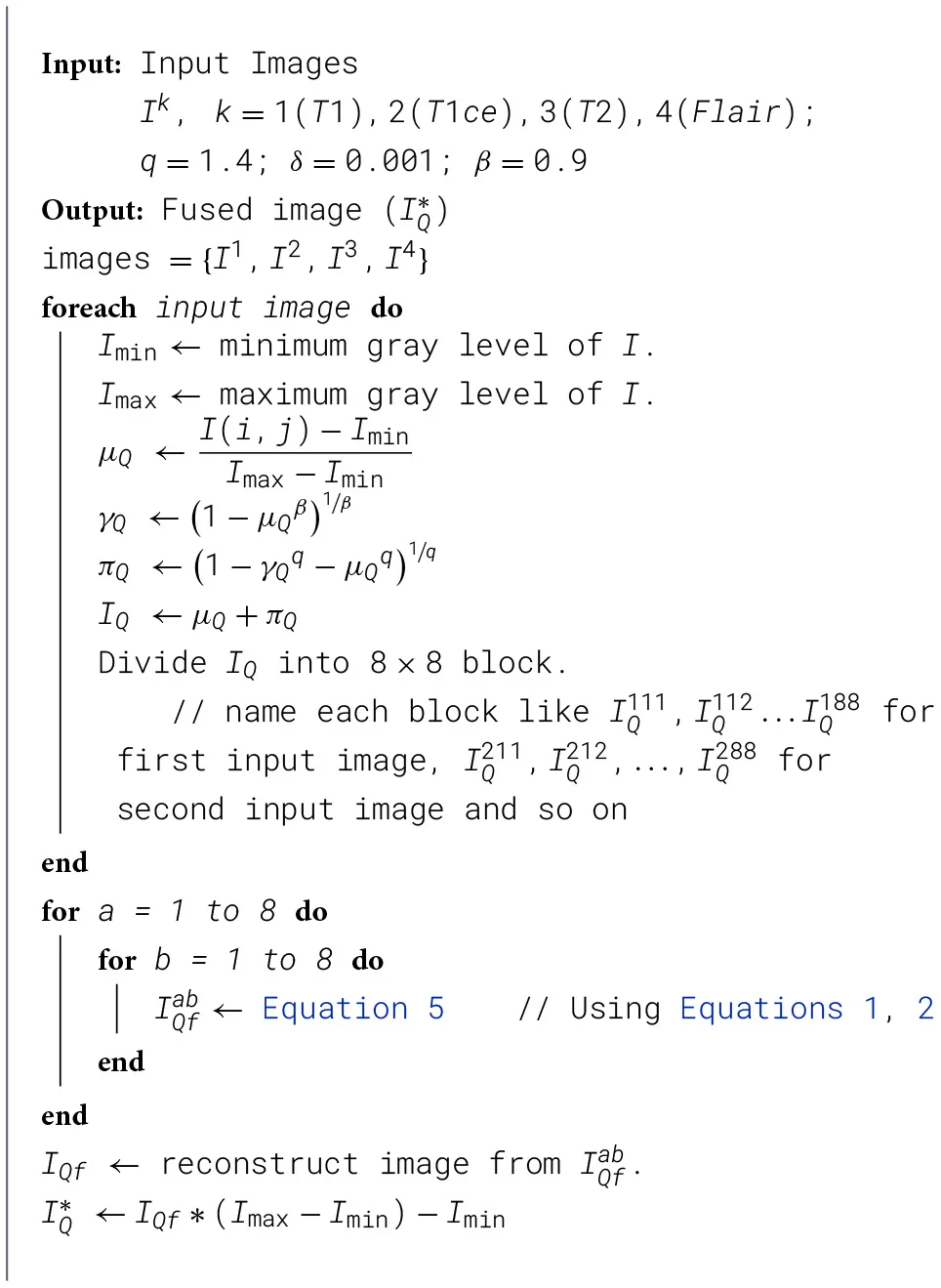

## 4 Experimental analysis and discussions

In this part, the experimental analysis of the proposed model is discussed in detail. Also, the performance evaluations are investigated. For the experimental analysis, the BraTS 2020 dataset is utilized, and the description of the dataset is discussed in Section 4.2.

### 4.1 Computational environments

The experimental evaluation was conducted on a system equipped with an Apple M2 processor featuring an 8-core CPU and an 8-core GPU. The machine operated on macOS Sequoia 15.3.1 and included 8 GB of unified memory with a memory bandwidth of 100 GB/s, along with a 256 GB solid-state drive (SSD). The proposed method, as well as all benchmark algorithms considered for comparison, was implemented and executed using MATLAB R2024a, and Python 3.12 (Python Software Foundation, Wilmington, DE, USA) was used for the segmentation process.

### 4.2 Data description

The experiments were conducted on the BraTS 2020 dataset, which comprises multi-modal MRI scans, including T1, T1ce, T2, and Flair images. The dataset provides comprehensive information about brain structures and tumors due to its multiple modalities. It contains a total of 494 MRI scans with brain tumors, primarily categorized into lower-grade glioma (LGG) and glioblastoma (GBM). In this experiment, 494 scans (369 training data + 125 validation) are analyzed across the four modalities. In this dataset, only the training data contains the segmentation mask. Each scan contains 155 slices covering the image from bottom to top. The analysis was performed using MATLAB R2024a software. All the images in the dataset are in the dimension of 240 × 240. The images can be obtained from https://www.kaggle.com/datasets/awsaf49/brats2020-training-data. A sample of ground truth segmentation image with not-tumor (class 0), non-ET (class 1), edema (class 2), and ET (class 4) is shown in Figure 2. In this experimental analysis, 250 images are considered for training, 45 for testing, and 74 for validation purposes.

**Figure 2:**
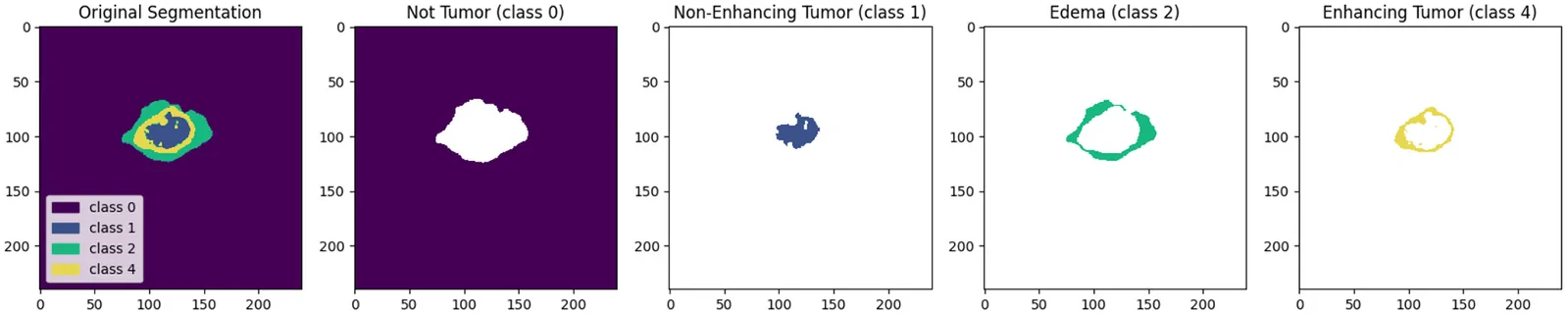
Segmented masks with different classes.

### 4.3 Implementation

In this part, the implementation of the proposed model is discussed in detail.

#### 4.3.1 Stage 1 — Fusion process

In stage 1, the process of the MMIF is employed as the following process.

**Step 1:** The multi-modal image dataset BraTS 2020 is considered as the input. The dataset contains four modalities, including T1, T1ce, T2, and FLAIR images of MRI.

**Step 2:** The images are fuzzified using Equation 9 for further process.

**Step 3:** The membership and non-membership of the images are determined by setting the parameter value β = 0.9. This process is done using the process given in subsection 2.3.

**Step 4:** The qROFI *I*^*k*^, *k* = 1, 2, 3, 4 are generated using Equation 10 for all the image modalities T1, T1ce, T2 and Flair, respectively. For this step, the value of *q* is fixed as *q* = 1.4.

**Step 5:** Each *I*^*k*^ for *k* = 1, 2, 3, 4 are decomposed into 64 blocks as 8 × 8 matrix.

**Step 6:** The decomposed blocks of each *I*^*k*^ for *k* = 1, 2, 3, 4 are fused using the fusion rule as given in Equation 8. For this fusion process, the qROF Hamacher t-norm and t-conorm operators are utilized as given in Equations 1, 2. In this step, the parameter δ value is considered as δ = 0.001 for the fusion process.

**Step 7:** Finally, the fused images are defuzzified using Equation 11 to obtain the output images.

Through this implementation, the fusion results are expected to be closely similar to the ground truth for the effective segmentation process. Further, these fused images are considered as the input data for the next stage to compute segmented images.

#### 4.3.2 Stage 2—Segmentation process

In stage 2, the fused images are applied to the segmentation process using the U-Net model, as processed based on the following settings.

In the dataset, each scan consists of 155 slices, which cover the image from top to bottom as described in the data description. In this study, 100 slices are considered from the 22nd slice for this segmentation process. In the UNet architecture, a combined loss function using the dice coefficient and cross-entropy is utilized. The Adam optimizer is employed with a learning rate of 0.001. For this work, the U-Net architecture comprises 23 convolution layers and follows a two-path design: an encoder and a decoder. Similar to traditional convolutional neural networks, the encoder extracts contextual information from the input using repeated combinations of convolution layers and max-pooling. Also, image dimensions are progressively decreased through downsampling. Each block of the encoder contains two 3 × 3 convolutional layers without padding, each followed by an activation function of rectified linear unit (ReLU) and a 2 × 2 max-pooling layer. The first convolutional layer set utilizes 64 filters, and subsequent layers double the number of filters at each step (e.g., 128 filters in the second set). This gradual increment in filters enhances feature extraction when spatial resolution decreases.

The decoder consists of the symmetric expandable pathway, which gradually restores the input spatial resolution while increasing the feature localization precision. At this stage, transposed convolution operations are used to upsample the feature representations in spatial dimensions. With each upsampling step, we reduce the depth of the feature maps by systematically halving the number of channels. The upsampled features are then concatenated with the corresponding encoder outputs through skip connections, which allows for effective integration of high-level semantic information with fine-grained spatial details. This fusion is followed by two successive 3 × 3 convolutional layers with ReLU activation to refine the features further. The final stage of the decoder is a 1 × 1 convolution layer that projects the refined feature maps onto two different cases, namely the ROI and the background. The segmentation outcome outlines the ROI, such as tumor regions, from surrounding tissues. Finally, to improve segmentation performance, a sigmoid activation function is employed at the output layer.

The U-Net architecture is a fully convolutional network as it avoids dense layers, enabling it to evaluate input images of any size. Skip connections are a fundamental feature of the model, connecting corresponding layers in the encoder and decoder paths. These connections enhance feature reuse, mitigate information loss, and stabilize training. Additionally, skip connections ensure a steady flow of gradients throughout the network. They effectively address vanishing gradient issues and facilitate better model performance and convergence.

### 4.4 Comparative and quantitative analyses

To show the effectiveness of the proposed model, it is compared with some extant image fusion frameworks including the method of SAM, MSVD (Naidu, 2011), PCA, IFIFS (Balasubramaniam and Ananthi, 2014), CBF (Shreyamsha Kumar, 2015), FFIF (Zhan et al., 2017), GIFS (Bavirisetti et al., 2017), CSMCA (Liu et al., 2019), GD (Kurban, 2023), and proposed model. Initially, the input multi-modal images were applied to the existing models, and fused images were obtained. Further, some important image quality metrics were determined for the fused images. For visual comparison, some sample images with different modalities, segmented masks, and their fused images with the aforementioned methods are displayed in Figures 3–5.

**Figure 3:**
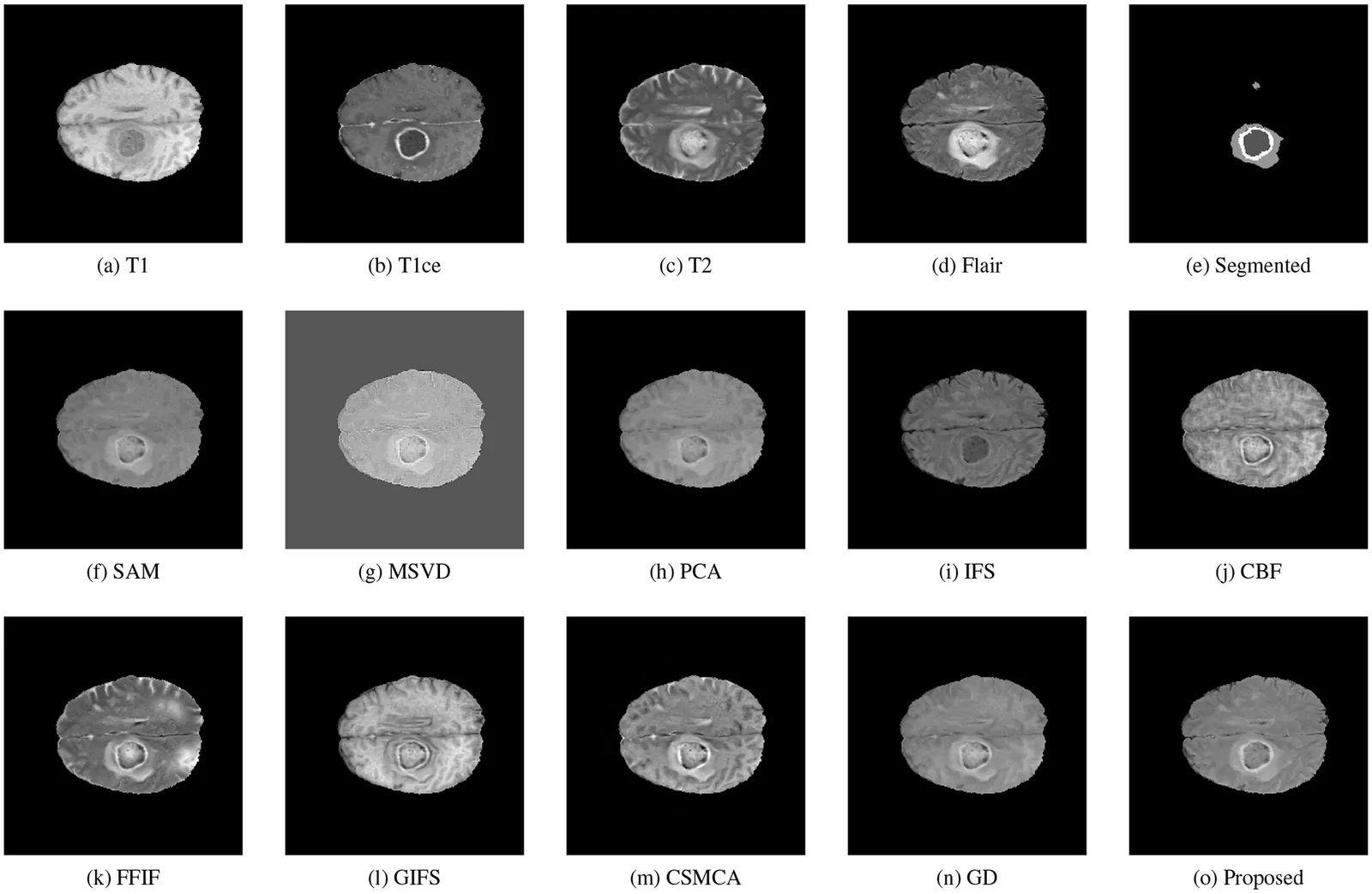
Sample 1 for visual comparison. **(a)** T1, **(b)** T1ce, **(c)** T2, **(d)** flair, **(e)** segmented, **(f)** SAM, **(g)** MSVD, **(h)** PCA, **(i)** IFS, **(j)** CBF, **(k)** FFIF, **(I)** GIFS, **(m)** CSMCA, **(n)** GD, and **(o)** proposed.

**Figure 4:**
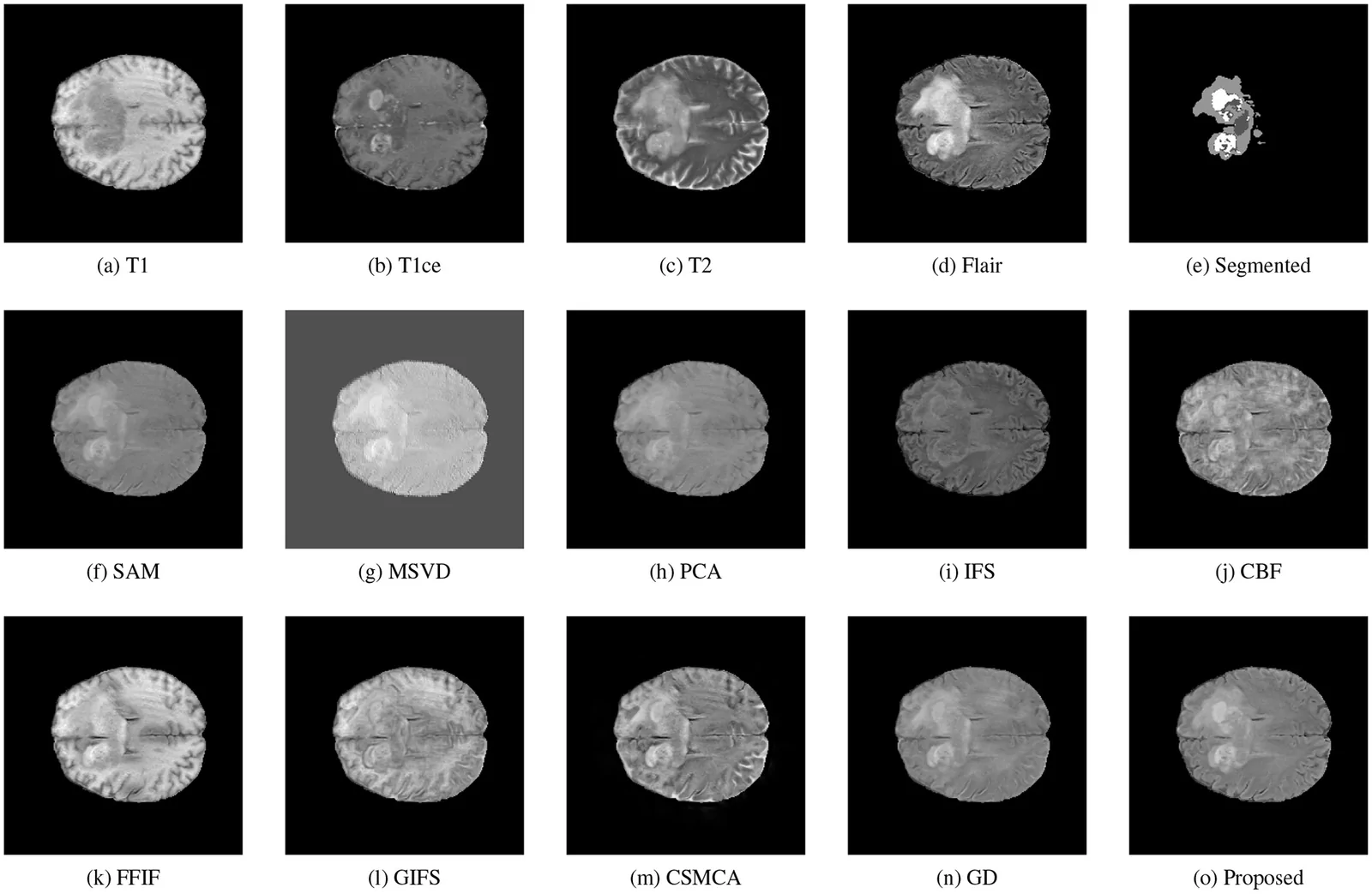
Sample 2 for visual comparison. **(a)** T1, **(b)** T1ce, **(c)** T2, **(d)** flair, **(e)** segmented, **(f)** SAM, **(g)** MSVD, **(h)** PCA, **(i)** IFS, **(j)** CBF, **(k)** FFIF, **(I)** GIFS, **(m)** CSMCA, **(n)** GD, and **(o)** proposed.

**Figure 5:**
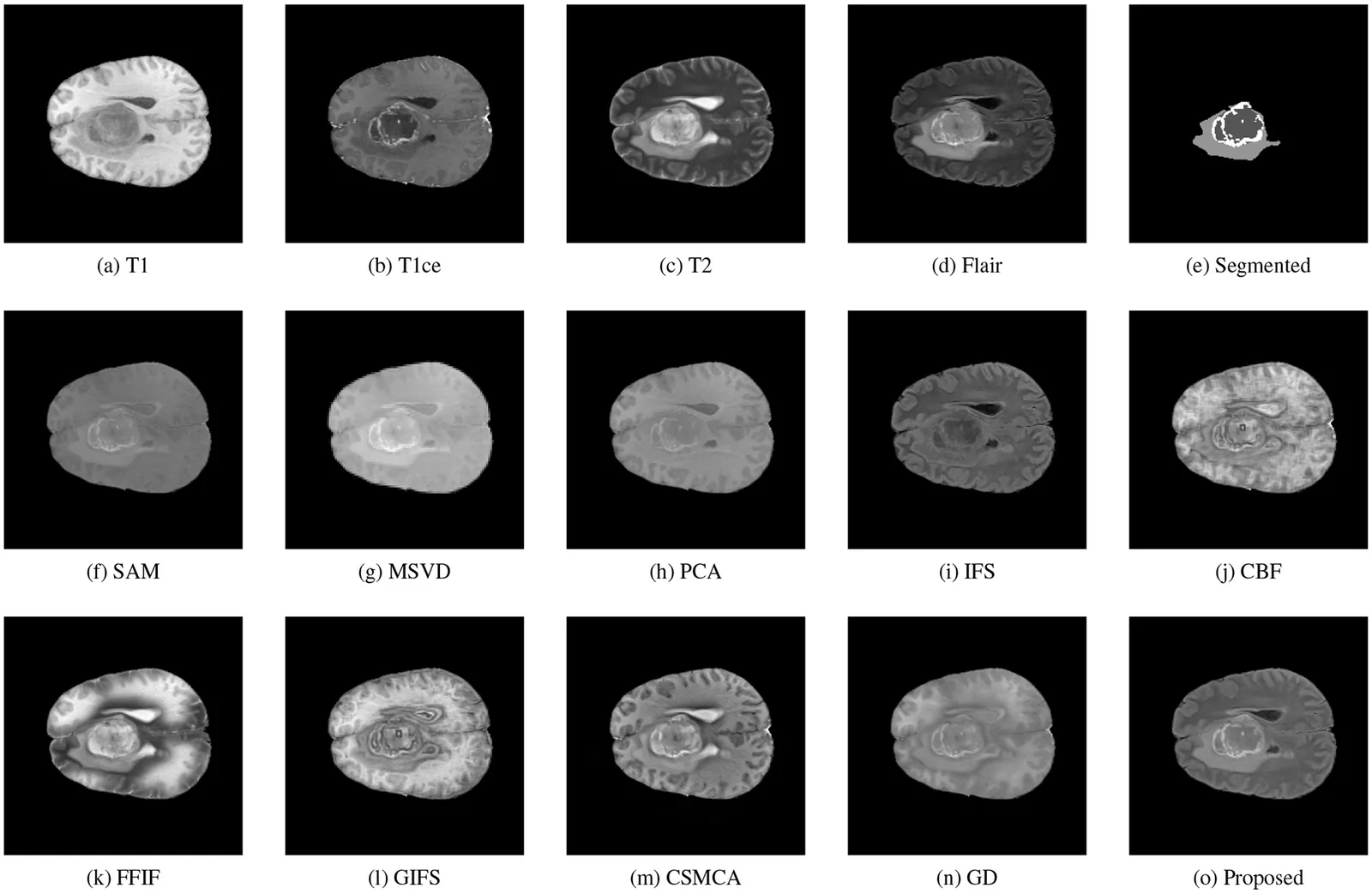
Sample 3 for visual comparison. **(a)** T1, **(b)** T1ce, **(c)** T2, **(d)** flair, **(e)** segmented, **(f)** SAM, **(g)** MSVD, **(h)** PCA, **(i)** IFS, **(j)** CBF, **(k)** FFIF, **(I)** GIFS, **(m)** CSMCA, **(n)** GD, and **(o)** proposed.

### 4.5 Performance metrics

The proposed model's performance was evaluated using seven mathematical metrics, including SSIM, MSSIM, PSNR, AMBE, correlation coefficient, MI, and UQI, respectively, discussed in the subsections 4.5.1–4.5.7, with the considered dataset, and quantified the average value with standard deviation in Table 1. These metrics serve as indicators of the fused image's quality in comparison to the input modality images and assess the ability of the model to retain information and improve image fusion quality. The following analysis elaborates on the results across these metrics:

**Table 1:** Results of performance measures between input modality images with fused image.

Models	Modality	SAM	MSVD	PCA	IFIFS	CBF	FFIF	GIFS	CSMCA	GD	Proposed
SSIM	T1	0.8537 ± 0.04	0.2855 ± 0.31	0.8993 ± 0.02	0.8018 ± 0.04	0.8262 ± 0.03	0.8116 ± 0.07	0.8549 ± 0.04	0.8300 ± 0.04	0.8505 ± 0.02	0.8111 ± 0.06
	T1ce	0.8976 ± 0.01	0.2713 ± 0.32	0.8960 ± 0.01	0.8509 ± 0.03	0.8123 ± 0.03	0.8061 ± 0.03	0.8100 ± 0.02	0.8153 ± 0.03	0.8720 ± 0.02	0.8703 ± 0.03
	T2	0.8539 ± 0.03	0.2429 ± 0.29	0.8190 ± 0.05	0.8400 ± 0.03	0.7970 ± 0.05	0.8568 ± 0.10	0.7717 ± 0.06	0.8276 ± 0.07	0.8475 ± 0.06	0.8709 ± 0.03
	Flair	0.8882 ± 0.03	0.2676 ± 0.29	0.8616 ± 0.05	0.8954 ± 0.02	0.8071 ± 0.04	0.8548 ± 0.07	0.7926 ± 0.03	0.8142 ± 0.04	0.8681 ± 0.04	0.9271 ± 0.01
MSSIM	T1	0.8478 ± 0.05	0.8410 ± 0.04	0.8982 ± 0.03	0.8090 ± 0.05	0.8610 ± 0.03	0.8043 ± 0.08	0.8951 ± 0.03	0.8300 ± 0.04	0.8526 ± 0.03	0.8154 ± 0.08
	T1ce	0.9119 ± 0.01	0.8589 ± 0.01	0.9182 ± 0.01	0.8958 ± 0.03	0.8482 ± 0.03	0.8144 ± 0.05	0.8449 ± 0.01	0.8415 ± 0.03	0.8867 ± 0.02	0.8978 ± 0.03
	T2	0.8669 ± 0.03	0.8166 ± 0.03	0.8264 ± 0.06	0.8191 ± 0.02	0.8096 ± 0.07	0.8531 ± 0.10	0.7668 ± 0.08	0.8693 ± 0.06	0.8548 ± 0.06	0.8753 ± 0.02
	Flair	0.9110 ± 0.03	0.8534 ± 0.04	0.8766 ± 0.07	0.9072 ± 0.02	0.8158 ± 0.09	0.8458 ± 0.09	0.7896 ± 0.08	0.8384 ± 0.05	0.8761 ± 0.07	0.9481 ± 0.01
PSNR	T1	17.5054 ± 1.69	16.4594 ± 3.40	19.3397 ± 1.22	14.4972 ± 1.30	19.3251 ± 1.10	17.6430 ± 4.60	21.4702 ± 2.32	17.7564 ± 1.60	18.1336 ± 1.29	16.8841 ± 2.17
	T1ce	24.3338 ± 1.10	13.2393 ± 1.81	22.1032 ± 1.01	26.9257 ± 0.65	20.3039 ± 1.45	19.3735 ± 3.12	18.3747 ± 1.07	20.7894 ± 0.56	22.8206 ± 0.52	24.1226 ± 0.74
	T2	22.6794 ± 1.60	13.4034 ± 1.86	20.8247 ± 2.42	20.6263 ± 1.19	19.8728 ± 3.15	21.0785 ± 5.55	17.9603 ± 3.59	22.0030 ± 2.40	22.0570 ± 2.84	22.1127 ± 1.01
	Flair	23.1331 ± 1.55	12.9949 ± 1.35	20.9022 ± 2.35	22.8672 ± 1.19	19.1593 ± 2.73	19.8838 ± 4.54	17.3408 ± 2.16	20.2623 ± 1.49	21.8204 ± 2.57	24.7130 ± 0.55
AMBE	T1	16.4245 ± 3.98	36.3351 ± 18.01	13.1588 ± 2.33	24.2125 ± 4.49	12.8842 ± 1.86	16.2630 ± 7.29	9.6448 ± 2.80	16.0628 ± 3.67	15.1602 ± 2.77	17.6024 ± 5.38
	T1ce	6.4992 ± 1.02	49.7834 ± 18.68	9.6342 ± 1.29	4.2855 ± 0.70	11.1404 ± 2.82	11.7328 ± 6.41	14.2066 ± 2.59	9.5410 ± 0.71	8.1299 ± 1.14	6.6779 ± 0.73
	T2	8.2576 ± 2.18	48.5269 ± 17.65	10.9540 ± 3.92	7.6168 ± 1.49	11.9718 ± 5.30	9.9959 ± 7.74	15.0547 ± 6.08	9.0328 ± 2.94	9.3393 ± 3.89	8.1006 ± 1.32
	Flair	8.3866 ± 2.11	49.9895 ± 15.31	11.2865 ± 3.96	6.4646 ± 1.77	13.0966 ± 5.73	10.5345 ± 7.75	15.8751 ± 5.14	10.5272 ± 2.58	9.9024 ± 3.91	7.0946 ± 0.80
Correlation	T1	0.9709 ± 0.01	0.9669 ± 0.01	0.9841 ± 0.01	0.9668 ± 0.01	0.9645 ± 0.01	0.9063 ± 0.05	0.9717 ± 0.01	0.9396 ± 0.01	0.9704 ± 0.01	0.9491 ± 0.03
	T1ce	0.9651 ± 0.01	0.9601 ± 0.01	0.9693 ± 0.01	0.9585 ± 0.01	0.9532 ± 0.01	0.8951 ± 0.05	0.9486 ± 0.02	0.9366 ± 0.01	0.9632 ± 0.01	0.9583 ± 0.02
	T2	0.9185 ± 0.04	0.9121 ± 0.04	0.8963 ± 0.06	0.8567 ± 0.05	0.8862 ± 0.07	0.8962 ± 0.07	0.8530 ± 0.09	0.9267 ± 0.04	0.9115 ± 0.06	0.9075 ± 0.03
	Flair	0.9382 ± 0.02	0.9331 ± 0.02	0.9197 ± 0.04	0.9101 ± 0.03	0.8823 ± 0.06	0.8789 ± 0.07	0.8636 ± 0.06	0.9029 ± 0.04	0.9201 ± 0.05	0.9583 ± 0.01
MI	T1	1.1640 ± 0.07	1.1663 ± 0.09	1.2710 ± 0.15	1.2126 ± 0.11	1.1498 ± 0.12	1.3154 ± 0.28	1.2120 ± 0.12	1.1845 ± 0.08	1.1327 ± 0.08	1.1559 ± 0.10
	T1ce	1.1190 ± 0.06	1.1153 ± 0.08	1.1476 ± 0.09	1.3148 ± 0.13	1.0888 ± 0.07	1.1635 ± 0.10	1.1063 ± 0.06	1.1160 ± 0.06	1.0929 ± 0.06	1.1395 ± 0.09
	T2	1.1452 ± 0.09	1.1561 ± 0.10	1.1356 ± 0.09	1.5202 ± 0.11	1.1311 ± 0.09	1.4271 ± 0.35	1.1576 ± 0.09	1.1821 ± 0.09	1.1355 ± 0.09	1.3159 ± 0.18
	Flair	1.2313 ± 0.09	1.2167 ± 0.09	1.1887 ± 0.10	1.3531 ± 0.13	1.1523 ± 0.07	1.3523 ± 0.14	1.1630 ± 0.06	1.1999 ± 0.08	1.1749 ± 0.09	1.4097 ± 0.16
UQI	T1	0.9408 ± 0.02	0.4262 ± 0.31	0.9635 ± 0.01	0.8612 ± 0.03	0.9668 ± 0.01	0.9279 ± 0.04	0.9787 ± 0.01	0.8296 ± 0.04	0.9522 ± 0.01	0.9224 ± 0.04
	T1ce	0.9749 ± 0.01	0.3247 ± 0.30	0.9603 ± 0.01	0.9808 ± 0.02	0.9489 ± 0.01	0.9457 ± 0.03	0.9323 ± 0.01	0.8408 ± 0.06	0.9657 ± 0.01	0.9767 ± 0.00
	T2	0.9608 ± 0.01	0.3377 ± 0.27	0.9491 ± 0.03	0.9551 ± 0.01	0.9445 ± 0.04	0.9523 ± 0.04	0.9310 ± 0.04	0.8457 ± 0.06	0.9593 ± 0.03	0.9703 ± 0.01
	Flair	0.9483 ± 0.04	0.3071 ± 0.26	0.9296 ± 0.04	0.9695 ± 0.02	0.9182 ± 0.06	0.9417 ± 0.05	0.8929 ± 0.07	0.8154 ± 0.05	0.9604 ± 0.07	0.9608 ± 0.03

#### 4.5.1 Structural Similarity Index Measure (SSIM)

SSIM evaluates how structurally similar the fused image is to the original images and ensures that the fusion process preserves essential structural details. As given in Equation 12, by calculating SSIM for each input image and the fused image, researchers can verify whether the fused image balances information from the source images. A high SSIM value between the fused image and an original image indicates that the fusion preserves perceptual qualities effectively.


$$\begin{array}{l} SSIM\left(O,F\right)=\frac{\left(2{\bar{\mu }}_{O}{\bar{\mu }}_{F}+c_{1}\right)\left(2\sigma_{OF}+c_{2}\right)}{\left({\bar{\mu }}_{O}^{2}+{\bar{\mu }}_{F}^{2}+c_{1}\right)\left(\sigma_{O}^{2}+\sigma_{F}^{2}+c_{2}\right)} \end{array}$$ (12)

where ${\bar{\mu }}_{O}$ and ${\bar{\mu }}_{F}$ represent the means of intensities of original (*O*) and fused (*F*), respectively. The measures σ_*O*_ and σ_*F*_ represent the variances of *O* and *F*, respectively. The notation *c*_1_ and *c*_2_ denote the constants that are added to ensure stability when ${\bar{\mu }}_{O}^{2}+{\bar{\mu }}_{F}^{2}$ and $\sigma_{O}^{2}+\sigma_{F}^{2}$ approaches to zero.

##### 4.5.1.1 SSIM

The SSIM values, presented in Table 1, show the structural similarity between the fused and input images. Higher SSIM indicates better quality and structure retention. The proposed framework consistently outperforms, achieving competitive or higher SSIM values for all modalities. The SAM yields better results due to direct mean. However, it does not indicate effective fusion quality.

#### 4.5.2 Multi-Scale Structural Similarity Index Measure (MSSIM)

MSSIM incorporates multi-resolution analysis as an extension of SSIM, offering enhanced adaptability and robustness. Since factors such as spatial resolution and sampling density impact the perceptual visibility of images, the single-scale SSIM formulation is often lacking to capture variations among different image scales. To overcome this, MSSIM computes similarity across multiple resolutions, enabling a comprehensive assessment of structural fidelity. It is defined as expressed in Equation 13:


$$\begin{array}{l} MSSIM\left(O,F\right)={\left[l_{n}\left(O,F\right)\right]}^{\alpha_{n}}\overset{n}{\underset{j=1}{\prod }}{\left[c_{j}\left(O,F\right)\right]}^{\beta_{j}}{\left[s_{j}\left(O,F\right)\right]}^{\gamma_{j}} \end{array}$$ (13)

where $l_{n}\left(O,F\right)=\frac{2{\bar{\mu }}_{O}{\bar{\mu }}_{F}+c_{1}}{{\bar{\mu }}_{O}^{2}+{\bar{\mu }}_{F}^{2}+c_{1}}$, $c_{j}\left(O,F\right)=\frac{2\sigma_{O}\sigma_{F}+c_{2}}{\sigma_{O}^{2}+\sigma_{F}^{2}+c_{2}}$, and $s_{j}\left(O,F\right)=\frac{\sigma_{OF}+c_{3}}{\sigma_{O}\sigma_{F}+c_{3}}$. Here, *l*_*n*_(*O, F*) computes luminance similarity at scale *n*, and *c*_*j*_(*O, F*) and *s*_*j*_(*O, F*) compute contrast and structural similarity at the *j*th scale, respectively.

The parameters α_*n*_, β_*j*_, and γ_*j*_ represent the relative contributions of luminance, contrast, and structural components in the overall similarity calculation. By aggregating information across multiple spatial resolutions, MSSIM presents a more reliable and perceptually stable image quality, particularly in complex fusion scenarios.

##### 4.5.2.1 MSSIM

Table 1 shows that the proposed model yields consistently high MSSIM scores across all imaging modalities, particularly for Flair and T2. Our model outperforms most existing techniques, including PCA and MSVD, while MSVD, CBF, and GIFS determine lower MSSIM scores. These findings underscore the effectiveness of our model in preserving multi-scale structural information.

#### 4.5.3 Peak Signal-to-Noise Ratio (PSNR)

PSNR is a significant metric to measure the quality of a fused image by comparing it to the original image. It evaluates how much noise or distortion has been introduced during the fusion process. A higher PSNR value demonstrates better improvement of the original image quality in the fused image. It can be calculated using Equation 14.


$$\begin{array}{l} PSNR\left(O,F\right)=10\log_{10}\left(\frac{\max{\left(O\left(i,j\right)\right)}^{2}}{MSE\left(O,F\right)}\right) \end{array}$$ (14)

where max(*O*(*i, j*)) denotes the maximum possible pixel intensity value in the image and *MSE*(*O, F*) denotes the Mean Squared Error (MSE) between the original image *O* and the fused image *F*. It is computed using Equation 15:


$$\begin{array}{l} MSE\left(O,F\right)=\frac{1}{mn}\overset{m}{\underset{i=1}{\sum }}\overset{n}{\underset{j=1}{\sum }}{\left(O\left(i,j\right)-F\left(i,j\right)\right)}^{2} \end{array}$$ (15)

Here, *m* and *n* denote the dimensions of the images, and *O*(*i, j*) and *F*(*i, j*) represent the pixel values of the original and fused images, respectively.

##### 4.5.3.1 PSNR

PSNR, a measure of signal fidelity, is reported in Table 1. Higher PSNR values indicate lower distortion and better image quality. The proposed model achieves competitive PSNR values across all modalities, notably outperforming MSVD in every case. For the Flair modality, it records a PSNR of 24.7130, surpassing PCA and several other methods. These results highlight the proposed model's effectiveness in preserving image clarity during fusion.

#### 4.5.4 Absolute Mean Brightness Error (AMBE)

AMBE measures brightness preserved in a fused image relative to the original image. It evaluates the absolute difference between their average intensity values, representing whether the fusion operation provides noticeable brightness variations. It is defined as in Equation 16.


$$\begin{array}{l} AMBE\left(O,F\right)=|{\bar{\mu }}_{O}-{\bar{\mu }}_{F}| \end{array}$$ (16)

where ${\bar{\mu }}_{F}$ and ${\bar{\mu }}_{O}$ represent the average intensity levels of the fused *F* and original images *O*, respectively.

##### 4.5.4.1 AMBE

Table 1 reflect the AMBE values between fused and source images. The proposed framework obtains low AMBE values across all modalities, with notably strong performance for Flair images, where it achieves 3.9791, showing negligible brightness deviation. MSVD yields higher AMBE values, showing its limited performance in preserving brightness fidelity.

#### 4.5.5 Correlation coefficient (r)

It indicates similarity between the images and computes the degree of linear association between the pixels. Scores close to 1 and –1 depict strong positive and negative correlation, respectively. The values near 0 indicate weak or negligible correlation. It can be measured using Equation 17:


$$\begin{array}{l} r\left(O,F\right)=\frac{\overset{M}{\underset{i=1}{\sum }}\overset{N}{\underset{j=1}{\sum }}\left(O\left(i,j\right)-Ō\right)\left(F\left(i,j\right)-\bar{F}\right)}{\sqrt{\overset{M}{\underset{i=1}{\sum }}\overset{N}{\underset{j=1}{\sum }}{\left(O\left(i,j\right)-Ō\right)}^{2}\overset{M}{\underset{i=1}{\sum }}\overset{N}{\underset{j=1}{\sum }}{\left(F\left(i,j\right)-\bar{F}\right)}^{2}}} \end{array}$$ (17)

where *O*(*i, j*) and *F*(*i, j*) represent pixel intensities at location (*i, j*), and Ō, $\bar{F}$ denote their respective mean values.

##### 4.5.5.1 Correlation

From Table 1, it is observed that our model consistently obtains high correlations across all modalities. This indicates strong linear agreement between the input and fused images.

#### 4.5.6 Mutual Information (MI)

It quantifies the amount of shared information between the original and fused images. It measures how effectively structural and textural details from the source images are preserved after fusion. Higher MI values show a better result. It can be measured by Equation 18:


$$\begin{array}{l} MI\left(O,F\right)=\underset{o}{\sum }\underset{f}{\sum }P\left(o,f\right)\log\left(\frac{P\left(o,f\right)}{P\left(o\right)P\left(f\right)}\right) \end{array}$$ (18)

where *P*(*o*) and *P*(*f*) represent the marginal probability density functions of *O* and *F*, respectively, and *P*(*o, f*) represents their joint probability distribution.

##### 4.5.6.1 MI

From Table 1, it is found that our model achieved competitive performance across all imaging modalities, maintaining maximum informational content from the source images. Although PCA and IFIFS yield marginally higher MI values, our model exhibits stable and balanced information preservation.

#### 4.5.7 Universal Quality Index (UQI)

UQI measures the quality of the fused image by considering contrast, luminance, and structural similarity compared to the original image. Higher UQI values show improved fusion performance. It is defined as given in Equation 19:


$$\begin{array}{l} UQI\left(O,F\right)=\frac{4\sigma_{OF}\left({\bar{\mu }}_{O}+{\bar{\mu }}_{F}\right)}{\left({\bar{\mu }}_{O}^{2}+{\bar{\mu }}_{F}^{2}\right)\left(\sigma_{O}^{2}+\sigma_{F}^{2}\right)} \end{array}$$ (19)

where ${\bar{\mu }}_{O}$ and ${\bar{\mu }}_{F}$ denote mean intensities, $\sigma_{O}^{2}$ and $\sigma_{F}^{2}$ represent variances, and σ_*OF*_ is the covariance between *O* and *F*.

##### 4.5.7.1 UQI

From Table 1, it is observed that our model consistently achieves higher UQI values across all modalities, particularly for Flair images with 0.9608. outperforming PCA, IFIFS, and CSMCA. This confirms its superiority in preserving contrast, luminance, and structural integrity.

#### 4.5.8 Overall quantitative evaluation

In summary, the quantitative results show that our model achieves improved fusion performance on all evaluation metrics. It frequently yields the highest average scores, representing the best trade-off between brightness consistency, structure preservation, and information retention. In particular, our model achieves better performance for Flair and T2 images, which outperforms the existing methods including MSVD, PCA, IFIFS, FFIF, CSMCA, GD, and SAM. Existing methods generally have limitations in image quality preservation, whereas our model ensures minimal distortion and maximum feature preservation. Hence, the proposed framework demonstrates its applicability for efficient medical image fusion.

### 4.6 Computation time analysis

Experimental results demonstrate that the proposed qROFS Hamachar t-norm- and t-conorm-based aggregation approach effectively fuses multimodal medical images. However, the analysis of execution time is significant for real-time deployment of the model. Therefore, a runtime analysis for the compared fusion models has been conducted and provided in Table 2. Although the proposed fusion framework consists of additional fuzzy membership construction and aggregation processes, the computational cost of the framework remains competitive when compared to other models. The average runtime is comparable to the simple fusion (SAM, PCA, and GD) and other fuzzy-based (FFI) methods, while substantially lower than other (MSVD, IFIFS, CBF, GIFS, and CSMCA) models. The proposed model incurs a moderately higher computational cost than the SAM, PCA, and GD methods due to the qROFS representation and the Hamacher aggregation processes. However, it is justified by the considerable improvements in the fusion quality and segmentation effectiveness.

**Table 2:** Runtime comparison.

Modality	SAM	MSVD	PCA	IFIFS	CBF	FFIF	GIFS	CSMCA	GD	Proposed
Run time (in s)	101	3429	115	17074	93973	1018	81333	4729498	862	3322

On the other hand, the segmentation stage employs the U-Net architecture, which benefits from GPU acceleration and allows efficient processing during inference. Therefore, the complete framework of the proposed fusion-segmentation approach is suitable for practical offline medical image analysis and computer-aided diagnosis systems.

### 4.7 Ablation study with the proposed qROFS-based fusion framework

The proposed framework has two sequential stages: qROFS-based multimodal image fusion and U-Net-based tumor segmentation. Since the U-Net architecture is adopted without any structural modifications, the principal methodological contribution of this work lies in the proposed qROFS fusion framework. Therefore, the ablation study is conducted to investigate the effectiveness of the proposed qROFS-based framework with alternative fuzzy sets, namely IFS, PFS, and FFS, as reported in Table 2. The obtained results clearly indicate that the proposed fusion scheme consistently achieves equal or superior performance across all considered evaluation metrics and imaging modalities. Additionally, a fuzzy set-based comparative analysis is performed by comparing IFS, PFS, and FFS with qROFS in the proposed fusion model to show the efficiency of the qROFS-based framework. Also, the results are demonstrated in Figures 6–8. From the comparison, it is observed that the images enhanced with IFS are too dark and under-enhanced. With FFS, the images are over-enhanced, and the tumor regions are faded or not clear. With qROFS, the model provides a better enhancement level when compared to IFS, PFS, and FFS, maintaining the tumor region highlighted and the non-affected region darker. This observation demonstrates the effectiveness of the qROFS compared to other fuzzy sets.

**Figure 6:**
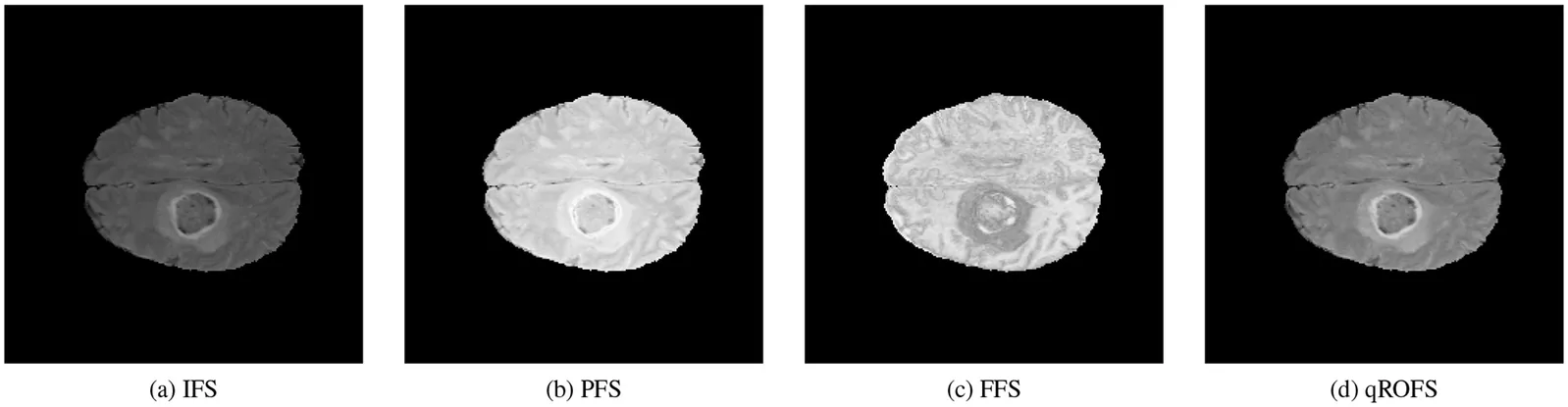
Sample 1 for fuzzy set comparison. **(a)** IFS, **(b)** PFS, **(c)** FFS, and **(d)** qROFS.

**Figure 7:**
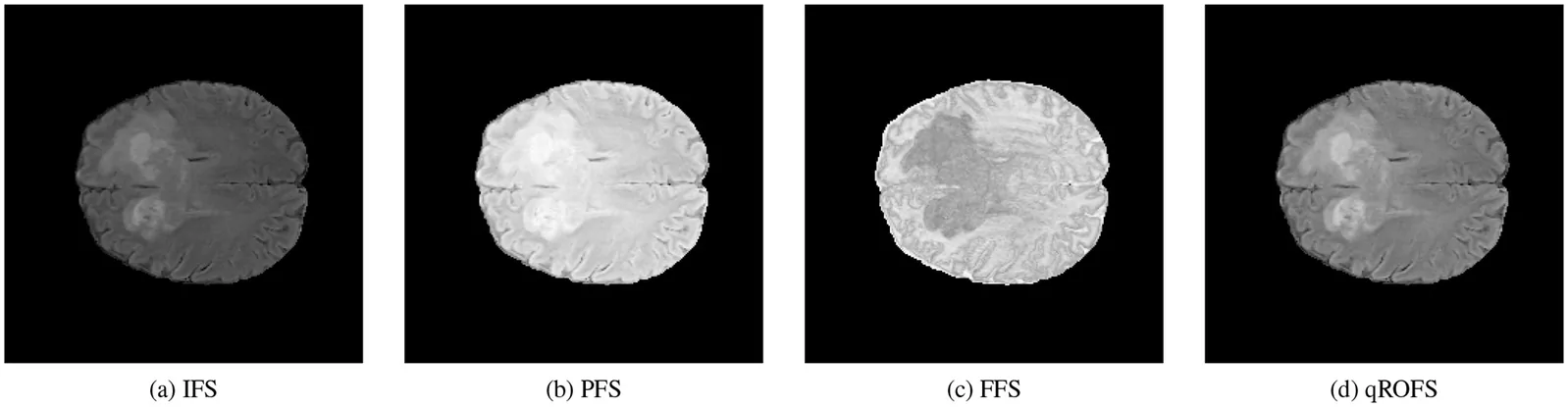
Sample 2 for fuzzy set comparison. **(a)** IFS, **(b)** PFS, **(c)** FFS, and **(d)** qROFS.

**Figure 8:**
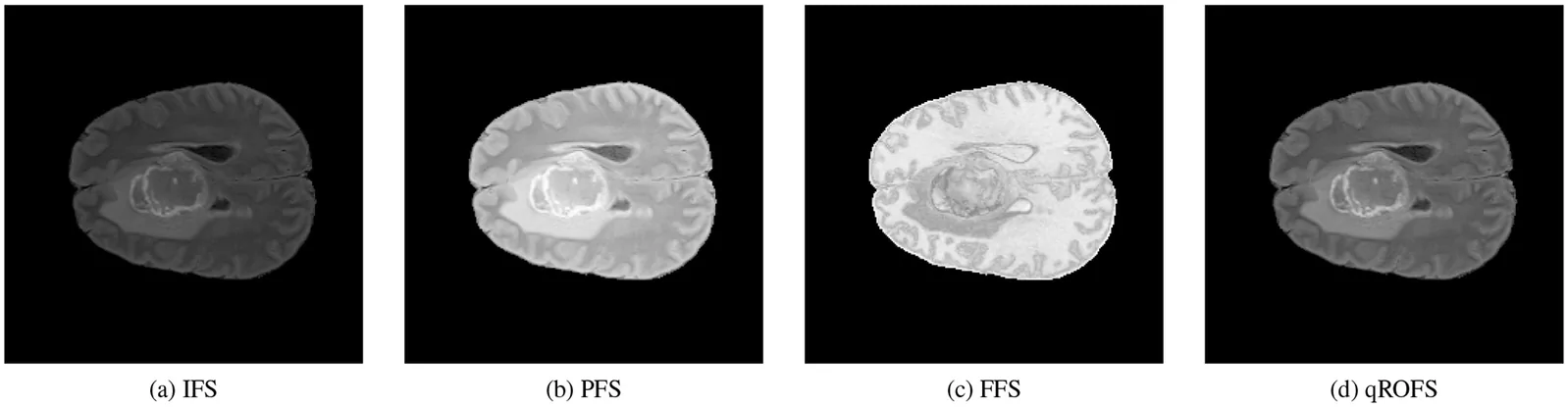
Sample 3 for fuzzy set comparison. **(a)** IFS, **(b)** PFS, **(c)** FFS, and **(d)** qROFS.

In SSIM evaluation, the proposed model shows a notable improvement, particularly for Flair images, where it outperforms others with 0.9271 ± 0.01, showing enhanced structural similarity preservation. In MSSIM evaluation (Table 3), our model achieves the highest score for Flair images (0.9481 ± 0.01), showing its capability to preserve both fine structural details and contrast consistency. The higher PSNR values achieved by our model, notably in Flair images (24.7130 ± 0.55), confirm its ability to offer high-quality fused images with minimal noise and distortion. The AMBE results further show that our model effectively preserves brightness characteristics, providing comparatively lower AMBE values than PFS and FFS, particularly in the T1ce modality. Additionally, correlation coefficient evaluations demonstrate the improved information retention capability of our model across all modalities. The MI analysis further supports this observation, showcasing that our method preserves a higher amount of complementary information from the input. Finally, the UQI results support the superior overall fusion quality, particularly for the T1ce modality (0.9767 ± 0.00), exceeding the performance of other models.

**Table 3:** Comparison of performance measures with various fuzzy set frameworks.

Models	Modality	IFS	PFS	FFS	Proposed
SSIM	T1	0.7849 ± 0.05	0.7910 ± 0.07	0.8568 ± 0.03	0.8111 ± 0.06
	T1ce	0.8760 ± 0.03	0.8012 ± 0.03	0.7754 ± 0.03	0.8703 ± 0.03
	T2	0.8593 ± 0.03	0.8170 ± 0.02	0.7198 ± 0.04	0.8709 ± 0.03
	Flair	0.9220 ± 0.03	0.8380 ± 0.02	0.7049 ± 0.05	0.9271 ± 0.01
MSSIM	T1	0.7506 ± 0.07	0.8372 ± 0.06	0.9008 ± 0.02	0.8154 ± 0.08
	T1ce	0.8929 ± 0.04	0.8061 ± 0.03	0.7894 ± 0.02	0.8978 ± 0.03
	T2	0.8441 ± 0.03	0.8149 ± 0.02	0.6910 ± 0.07	0.8753 ± 0.02
	Flair	0.9367 ± 0.02	0.8417 ± 0.02	0.6679 ± 0.07	0.9481 ± 0.01
PSNR	T1	13.2703 ± 1.32	20.3003 ± 1.90	21.2195 ± 1.30	16.8841 ± 2.17
	T1ce	24.7379 ± 1.71	13.0399 ± 0.65	13.2117 ± 0.99	24.1226 ± 0.74
	T2	20.5830 ± 1.29	13.3647 ± 0.80	13.3008 ± 1.98	22.1127 ± 1.01
	Flair	23.6450 ± 2.51	13.0336 ± 0.42	12.4793 ± 1.62	24.7130 ± 0.55
AMBE	T1	27.9482 ± 5.37	11.1001 ± 2.88	7.9188 ± 1.46	17.6024 ± 5.38
	T1ce	6.6026 ± 1.95	29.0350 ± 2.94	28.2904 ± 4.91	6.6779 ± 0.73
	T2	27.8898 ± 3.70	26.6059 ± 7.59	8.1006 ± 1.32	8.1006 ± 1.32
	Flair	29.2473 ± 2.97	29.9533 ± 7.89	7.0946 ± 0.80	7.0946 ± 0.80
Correlation	T1	0.9470 ± 0.03	0.9664 ± 0.02	0.9687 ± 0.01	0.9491 ± 0.03
	T1ce	0.9572 ± 0.02	0.9621 ± 0.01	0.9414 ± 0.02	0.9583 ± 0.02
	T2	0.9056 ± 0.03	0.9070 ± 0.03	0.8542 ± 0.07	0.9075 ± 0.03
	Flair	0.9603 ± 0.01	0.9282 ± 0.01	0.8188 ± 0.03	0.9583 ± 0.01
MI	T1	1.1089 ± 0.09	1.1717 ± 0.13	1.4029 ± 0.19	1.1559 ± 0.10
	T1ce	1.1036 ± 0.09	1.1519 ± 0.11	1.1246 ± 0.09	1.1395 ± 0.09
	T2	1.2670 ± 0.18	1.3467 ± 0.20	1.1989 ± 0.11	1.3159 ± 0.18
	Flair	1.3757 ± 0.16	1.3893 ± 0.15	1.1980 ± 0.10	1.4097 ± 0.16
UQI	T1	0.8166 ± 0.04	0.9754 ± 0.01	0.9717 ± 0.01	0.9224 ± 0.04
	T1ce	0.9533 ± 0.02	0.8444 ± 0.02	0.8434 ± 0.03	0.9767 ± 0.00
	T2	0.9338 ± 0.02	0.8561 ± 0.04	0.8606 ± 0.05	0.9703 ± 0.01
	Flair	0.9738 ± 0.01	0.8233 ± 0.04	0.8242 ± 0.05	0.9608 ± 0.03

Overall, the proposed fusion strategy demonstrates its robustness in preserving critical contrast, structural, and informational features while reducing degradation issues, ensuring its applicability and reliability for advanced medical image fusion applications.

### 4.8 Evaluation results using confusion matrix

The performance of the segmentation framework is evaluated by the confusion matrix, which helps to summarize the classification results by comparing the predicted labels with ground truth labels. It consists of four main components as follows:

- *TP*—number of correctly classified positive instances,
- *TN*—number of correctly classified negative instances,
- *FP*—number of incorrectly classified negative instances,
- *FN*—number of incorrectly classified positive instances.

The components offer the basis for calculating several widely utilized evaluation metrics, including accuracy, precision, sensitivity, and specificity, using Equations 20–23, respectively. These performance metrics help to assess the robustness of the framework. Based on the confusion matrix, the performance metrics are computed as follows:

**Accuracy:** It evaluates the overall classification rate and is given by:


$$\begin{array}{l} \text{Accuracy}=\frac{TP+TN}{TP+TN+FP+FN} \end{array}$$ (20)

**Precision:** It calculates the proportion of positive predictions made by the classifier that are true:


$$\begin{array}{l} \text{Precision}=\frac{TP}{TP+FP} \end{array}$$ (21)

**Sensitivity:** It calculates the percentage of positive predictions that are correct:


$$\begin{array}{l} \text{Sensitivity}=\frac{TP}{TP+FN} \end{array}$$ (22)

**Specificity:** It computes the percentage of negative instances that are true:


$$\begin{array}{l} \text{Specificity}=\frac{TN}{TN+FP} \end{array}$$ (23)

The results of the performance evaluation metrics such as Loss, Accuracy, MeanIOU, Dice coefficient, Precision, Sensitivity, and Specificity are depicted in Table 4.

**Table 4:** Results of performance metrics.

Metrics	Results
Accuracy	0.9896
Precision	0.9895
Sensitivity	0.9894
Specificity	0.9965
Loss	0.4093
MeanIOU	0.8597
Dice coefficient	0.6533

The experimental results confirm that the proposed framework successfully achieves the objectives indicated in this study.

(i) The qROFS-based fusion model effectively combines complementary information from the four MRI modalities of the BraTS2020 dataset. This achievement is supported by the competitive performance (Table 1) of the evaluation metrics, including SSIM, MSSIM, PSNR, Correlation, MI, and UQI.
(ii) The construction of qROFIs before the fusion process ensures the images address the uncertainty associated with medical image information.
(iii) The comparative analysis with both existing fusion methods and alternative fuzzy set frameworks demonstrates the capability of the proposed t-norm and t-conorm operators-based approach to preserve structural information, maintain image quality, and preserve diagnostically relevant information.
(iv) The high segmentation accuracy determined using the U-Net model further demonstrates that the fused images provide informative representations for reliable brain tumor delineation.

In summary, these results support the scientific claims of the proposed method and demonstrate that the established research objectives have been accomplished.

### 4.9 Clinical interpretability and practical applicability

The proposed model is designed to act as a computer-aided decision support system rather than an alternative to clinical expertise. It improves the visualization of tumor-related features by fusing complementary information from multiple MRI modalities, including T1, T1ce, T2, and Flair. Instead of examining multiple modalities at the same time, it enables radiologists to analyze a single informative image, significantly preserving clinically meaningful information and reducing interpretation time.

On the other hand, the segmentation using the U-Net approach outlines the tumor region from the fused image. It offers an initial assessment of tumor boundaries to help clinicians during diagnosis, treatment planning, and disease monitoring. Since the results are computed from information given by multiple MRI modalities, they can offer more comprehensive tumor localization than single modality-based analyses.

The proposed framework can be integrated into clinical radiology workflows as a post-processing step after MRI acquisition in practical environments. The fused images and segmentation masks can be accessible to radiologists through the picture archiving and communication system (PACS) or similar systems for expert review before diagnosis. This framework aims to enhance visualization and reduce manual interpretation, while maintaining the clinician as the ultimate decision maker.

## 5 Conclusions, limitations, and future work

The present study proposed a qROFS-based MMIF framework with a U-Net-driven BTS model. Initially, the model generates the corresponding qROF representation of four distinct input MRI brain modalities to enhance contrast and structural visibility. Further, the qROF Hamacher t-norm- and t-conorm-operator-based fusion framework combines the complementary pixel-level information across modalities, producing improved fused images that are well-suited for the subsequent segmentation process. The U-Net model is applied to effectively extract tumor regions from the fused representations, enabling accurate and reliable representation. By addressing the challenges posed by uncertainty, noise, and incomplete spatial information in medical imaging, the proposed framework provides a comprehensive solution for complex diagnostic tasks.

The experiments on the BraTS 2020 dataset show significant improvements in fusion quality and segmentation accuracy, which confirm the ability of the framework to capture clinically relevant features. The findings from the fusion framework show better preservation of tumor characteristics and anatomical structures when compared to existing methods, while the fuzzy-set-based ablation study further confirms the effectiveness of the proposed qROFS-driven strategy. Moreover, the segmentation performance obtained by using U-Net validates the suitability of the fused images for accurate tumor localization and boundary detection, which reinforces the reliability of the framework. Overall, the experimental evaluation proves that the proposed framework meets the primary objectives of this work.

The present study outlines the importance of integrating advanced fuzzy modeling approaches with modern deep learning architectures to achieve robust and adaptive medical image analysis. Such frameworks greatly enhance reliability, support clinical interpretations, and contribute to more informed treatment planning, providing meaningful advantages in real-world healthcare environments.

### 5.1 Limitations

Although the proposed model offers effective performance, it has some limitations as follows:

(i) In the qROFI construction, the parameter *q* was obtained by trial-and-error experiments.
(ii) The fusion process was designed using the Hamachar t-norm and t-conorm. The results may differ if other operators are employed.
(iii) Experimental analysis was conducted only on the BraTS2020 dataset.
(iv) The proposed model was only evaluated on MRI image modalities due to its main objective. Its generalization was not evaluated on CT, PET, SPECT, etc.
(v) The quantitative performance metrics were evaluated without direct clinical interpretation by medical specialists, involving radiologists and multi-center clinical datasets.

### 5.2 Future work

To address the aforesaid limitations, this study can be further extended as follows:

(i) The selection of the parameter *q* plays a significant role in balancing membership and non-membership information, and its optimal tuning using metaheuristic optimization or adaptive search algorithms constitutes an important future research direction.
(ii) The utilization of flexible aggregation operators based on various t-norms and t-conorms may further strengthen the fusion process by enabling dynamic control over the integration of multi-modal information, thereby preserving salient features while suppressing noise and redundancy.
(iii) Although the proposed model was validated on the widely adopted BraTS2020 benchmark dataset to ensure a fair comparison with existing methods, further evaluation on more recent BraTS releases and other publicly available multimodal brain MRI datasets will be considered in future work. Such cross-dataset validation will offer additional evidence of the generalization capability and robustness of the proposed framework under diverse imaging conditions.
(iv) Due to its main objective, the proposed model was evaluated on MRI image modalities. Future work will generalize the model for other modalities, such as CT, PET, SPECT, etc.
(v) The objective of this study is to develop and evaluate a computational framework rather than to establish clinical diagnostic guidelines. Therefore, the quantitative performance metrics were evaluated without direct clinical interpretation by medical specialists. Future work will involve collaboration with radiologists and neuro-oncology experts to assess the clinical relevance of the fused images and segmentation results, thereby providing additional validation of the proposed framework in practical diagnostic settings.

Future work can be focused on the implementation of advanced AI models and hybrid deep learning architectures for segmentation. Particularly,

(i) Incorporating transformer-based networks and attention-driven strategies to model long-range spatial dependencies can enhance global feature delineation and contextual understanding.
(ii) Graph-based attention models, integrated with the U-Net backbone, can be employed to capture complex inter-regional relationships within medical images. By modeling anatomical structures as interconnected graph components, such approaches facilitate enhanced context-aware segmentation, which is particularly beneficial for accurately delineating heterogeneous tumor regions.
(iii) Future work will also be focused on uncertainty-aware segmentation by using Bayesian deep learning, Monte Carlo Dropout, ensemble learning, or evidential learning to estimate the confidence of the prediction. These approaches can produce voxel-wise uncertainty maps that can help clinicians to evaluate the reliability of an automatic tumor segmentation.

## Data Availability

The original contributions presented in the study are included in the article/supplementary material, further inquiries can be directed to the corresponding author/s.
